# Composition of mucus- and digesta-associated bacteria in growing pigs with and without diarrhea differed according to the presence of colonic inflammation

**DOI:** 10.1186/s12866-023-02874-1

**Published:** 2023-05-20

**Authors:** Farhad M. Panah, Charlotte Lauridsen, Ole Højberg, Henrik Elvang Jensen, Tina Skau Nielsen

**Affiliations:** 1 Department of Animal and Veterinary Sciences, Aarhus University, Tjele, Denmark; 2 Department of Veterinary and Animal Sciences, University of Copenhagen, Copenhagen, Denmark

**Keywords:** Gut health, Pig gut microbiota, Colonic inflammation, Colitis, Diarrhea, Growing diarrhea, Ulcerative colitis

## Abstract

**Background:**

In the pig production, diarrhea can occur during different growth stages including the period 4–16 weeks post weaning, during which a diarrheal outbreak also termed as colitis-complex diarrhea (CCD) can occur and it is distinct from post-weaning diarrhea (1–2 weeks post weaning). We hypothesized that CCD in growing pigs is associated with changes in colonic microbiota composition and fermentation patterns, and the aim of the present observational study was to identify changes in digesta-associated bacteria (DAB) and mucus-associated bacteria (MAB) in the colon of growing pigs with and without diarrhea. A total number of 30 pigs (8, 11, and 12 weeks of age) were selected; 20 showed clinical signs of diarrhea and 10 appeared healthy. Based on histopathological examination of colonic tissues, 21 pigs were selected for further studies and classified as follows: without diarrhea, no colon inflammation (NoDiar; *n* = 5), with diarrhea, without colonic inflammation (DiarNoInfl; *n* = 4), and with diarrhea, with colonic inflammation (DiarInfl; *n* = 12). Composition (based on 16S rRNA gene amplicon sequencing) and fermentation pattern (short-chain fatty acids; SCFA profile) of the DAB and MAB communities were characterized.

**Results:**

The DAB showed higher alpha diversity compared to MAB in all pigs, and both DAB and MAB showed lowest alpha diversity in the DiarNoInfl group. Beta diversity was significantly different between DAB and MAB as well as between diarrheal groups in both DAB and MAB. Compared to NoDiar, DiarInfl showed increased abundance of various taxa, incl. certain pathogens, in both digesta and mucus, as well as decreased digesta butyrate concentration. However, DiarNoInfl showed reduced abundance of different genera (mainly *Firmicutes*) compared to NoDiar, but still lower butyrate concentration.

**Conclusion:**

Diversity and composition of MAB and DAB changed in diarrheal groups depending on presence/absence of colonic inflammation. We also suggest that DiarNoInfl group was at the earlier stage of diarrhea compared with DiarInfl, with a link to dysbiosis of colonic bacterial composition as well as reduced butyrate concentration, which plays a pivotal role in gut health. This could have led to diarrhea with inflammation due to a dysbiosis, associated with an increase in e.g., *Escherichia-Shigella* (*Proteobacteria*), *Helicobacter (Campylobacterota),* and *Bifidobacterium (Actinobacteriota)*, which may tolerate or utilize oxygen and cause epithelial hypoxia and inflammation. The increased consumption of oxygen in epithelial mucosal layer by infiltrated neutrophils may also have added up to this hypoxia. Overall, the results confirmed that changes in DAB and MAB were associated with CCD and reduced butyrate concentration in digesta. Moreover, DAB might suffice for future community-based studies of CCD.

**Supplementary Information:**

The online version contains supplementary material available at 10.1186/s12866-023-02874-1.

## Background

In the pig production, diarrhea can occur during different growth stages including the period 4–16 weeks post weaning. During this period, diarrhea has also been termed as colitis-complex diarrhea (CCD) being distinct from post-weaning diarrhea occurring 1–2 weeks post weaning, which is mainly caused by a small intestinal infection with an enterotoxigenic *E. coli* [1]. Colitis-complex diarrhea is multifactorial with a vague etiology, often associated with colonic inflammation due to the presence of specific pathogens, e.g. *Brachyspira* (*B*.) *pilosicoli*, and/or dietary factors [2]. Moreover, *B. hyodysenteriae* [3]*, B. pilosicoli* [4]*, Salmonella (S.) enterica* serovar Typhimurium [5], *Escherichia (E.) coli.* [1], swine whipworms [6], and in some cases *Lawsonia (L.) intracellularis* [7, 8] have been reported to be involved in the etiology of CCD. Previous studies showed that not only pathogens but also other factors could cause diarrhea in growing pigs [9], including social and physical stresses, e.g. mixing pigs with non-littermates, and reduced room temperature at weaning [10]. This simply reflects the complexity of CCD etiology in growing pigs.

The large intestine of pigs is dominated by a diverse number of different microbes [11], which are involved in harvesting energy from undigested feedstuffs [12], producing short-chain fatty acids (SCFA) and training the host immune system [13]. Changes in the gut microbiota composition is, to a large extent, a reflection of changes in the diet [14] and the health status of the host [15]. Carbohydrate-fermenting bacteria (e.g. SCFA-producing bacteria), such as the family of *Lachnospiraceae* (in particular *Roseburia* spp.)*,* and the genera *Prevotella* and *Ruminococcus*, take part in maintaining gut health by e.g. producing butyrate [16]. Fermentation products of gut bacteria, such as butyrate, are sources of energy for colonocytes in addition to glucose and glutamine from vascular origin [17]. Especially, butyrate confers remarkable beneficial effects to the host through inhibition of inflammation, reinforcing various components of the colonic defense barrier and decreasing oxidative stress [18]. Butyrate is the preferred substrate for colonocyte metabolism [19] and may have selective antimicrobial effects [20]. Therefore, gut microbiota is expected to be an informative phenotype of the animal to be studied aimed at understanding the influence of changes in gut microbiota on the host, the consequent changes in the chemical production of fermentation and potentially demystifying the etiology of CCD in growing pigs.

Although mucus-associated bacteria (MAB) are in closer proximity to the host’s intestinal epithelial cells when compared to digesta-associated bacteria (DAB) [21], there is a sparse body of literature investigating the structural changes of MAB community in growing pigs with CCD [14]. The intimate contact of MAB with the host, pronounces these communities more important than luminal bacteria, in terms of affecting host physiological and functional status [21]. The difference in microbial ecosystems between digesta and mucus, e.g. caused by a decline in oxygen availability from mucus to digesta [22] and differences in substrate availability, such as mucin glycoproteins, makes DAB and MAB compositions potentially distinct with different characteristics. To better understand the influence of colonic microbiota on the occurrence of CCD, it is of importance to closely scrutinize both the DAB and MAB in growing pigs.

We hypothesized that the DAB and MAB communities, as well as the fermentation pattern of colonic microbiota, would change in association with the incidence of CCD. The objective of this study was, therefore, to understand the changes in composition of DAB and MAB as well as their metabolites in healthy and diarrheic growing pigs to cast light on the etiology of CCD.

## Results

### Clinical and postmortem diagnosis of CCD

**Fig. 1:**
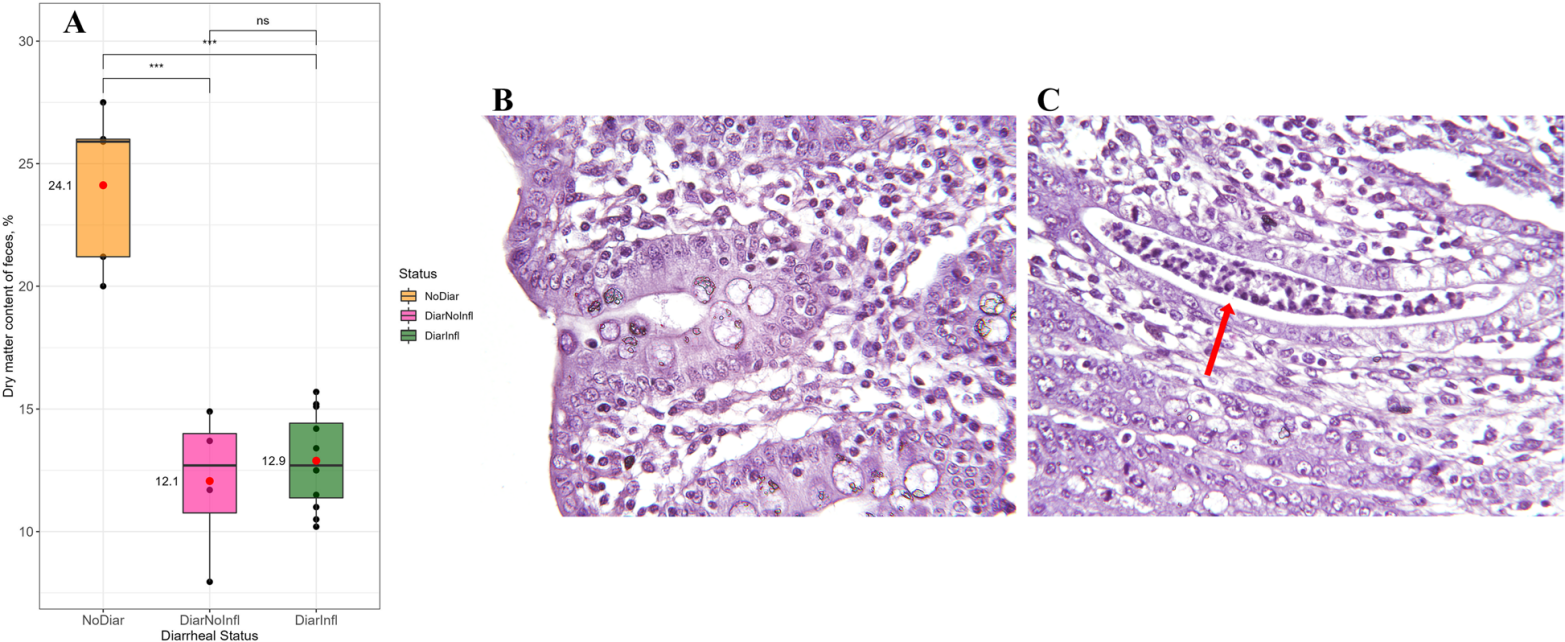
Dry matter content (%) of fecal samples from the three different groups **A**. Comparison of means was done by an unpaired student t-test. A representative histology-stained section of Co2 for NoDiar group with × 25 focal magnification **B** and infiltration of inflammatory cells between and within crypts of Co2 (red arrow) for DiarInfl group with × 25 focal magnification **C**

After inspection of the randomly chosen pens (*n* = 10), 20 pigs were assessed as ‘diarrheic’ and 10 pigs as ‘clinically healthy controls’. The exact number of animals in each group in regard with different parameters are presented in Tables S1 and Table S2 indicates the number of samples in each groups per different types of analysis. Diarrheic pigs had watery and lose diarrhea with shiny mucus on the stool and they were pronounced diarrheic if fecal DM was < 18% (Fig. 1A). The NoDiar group had significantly higher dry matter content of stool compared to diarrheal groups (24.1 vs. 12.5%). Histological examination failed for nine pigs; hence all the results are from 21 animals since we used histology as the benchmark of our diagnosis. Based on our histological results, pigs were classified as healthy controls (Fig. 1B) without clinical and postmortem signs of diarrhea and inflammation (NoDiar; *n* = 5), diarrheal without colonic inflammation (DiarNoInfl; *n* = 4), and diarrheal with inflammation (DiarInfl, Fig. 1C; *n* = 12). None of the tested pigs showed shedding of specific pathogens, e.g. *B. hyodysenteriae*, *B. pilosicoli* and *L. intracellularis*, in the stool.

### Microbial fermentation products

Table 1 shows the pH and concentration (mmol/kg digesta) of SCFA in digesta recovered from Co2 and Co3 of the different groups. Among all SCFAs, only butyrate was affected by gender, it was lower in females than in males; 12.4 vs. 8.80 mmol/kg digesta, respectively. Sample type had no effect on pH and SCFA concentration; however, since the estimate for sample type was not ignorable relative to other factors, the results are presented for both Co2 and Co3. Concentration of total SCFA and pH of digesta were not different among groups, while the concentration of individual SCFA such as butyrate, valerate and iso-acids differed between groups. Compared to NoDiar, DiarInfl showed on average 36.4% less butyrate concentration in Co2; and in Co3, DiarNoInfl and DiarInfl had on average 41.2% less butyrate compared with NoDiar. Valerate, and iso-acids were also lower in two groups with diarrhea, compared with NoDiar group.

**Table 1:** Digesta pH and concentration of SCFA (mmol/kg wet sample) in Co2 and Co3

	Groups^1^		
	NoDiar	DiarNoInfl	DiarInfl
Co2			
pH	6.0 (5.75–6.36)	6.0 (5.64–6.31)	6.20 (6.02–6.48)
SCFA^2^	118 (96.9–145)	112 (91.1–137)	111 (93.9–131)
Butyrate	15.1 (10.0–22.7) ^b^	10.0 (6.55–15.3) ^ab^	9.60 (6.86–13.5) ^a^
Propionate	29.2 (22.6–37.8)	28.2 (21.7–36.7)	28.0 (22.5–34.9)
Acetate	69.1 (58.5–81.7)	69.1 (58.4–81.8)	70.9 (63.0–80.3)
Valerate	3.50 (1.80–6.81)	3.10 (1.53–6.18)	2.1 (1.17–3.79)
Iso-acids	1.70 (1–2.8) ^b^	0.70 (0.42–1.22) ^a^	1.0 (0.63–1.58) ^a^
Co3			
pH	6.40 (6.04–6.69)	6.30 (5.90–6.74)	6.40 (6.18–6.61)
SCFA	108 (88.0–131.5)	96.3 (76.6–121)	101 (85.6–119)
Butyrate	13.6 (9.03–20.6) ^b^	7.40 (4.60–11.9)^a^	8.60 (6.19–12.0) ^a^
Propionate	23.6 (18.2–30.5)	22.0 (16.4–29.5)	23.8 (19.2–29.5)
Acetate	65.0 (54.9–77.0)	63.6 (52.1–77.6)	66.2 (58.6–74.8)
Valerate	3.20 (1.66–6.32) ^b^	1.70 (0.79–3.70) ^ab^	1.80 (1.02–3.27) ^a^
Iso-acids	2.30 (1.39–3.96) ^c^	0.70 (0.39–1.30) ^a^	1.20 (0.77–1.90) ^b^

^1^Diarrheal groups: no diarrheal control (NoDiar; *n* = 10), diarrheal without inflammation in colon (DiarNoInfl; *n* = 6), and diarrheal with inflammation in colon (DiarInfl; *n* = 22)

^2^SCFA: Short-chain fatty acids (mg/kg wet sample). Estimated Marginal Means are reported with their 95% confidence intervals and rows with different superscript letters indicate different EMMs (*P* < 0.05) with pairwise comparison adjusted by BH

Concentrations of biogenic amines were not significantly different between two segments of colon (Table 2). The NoDiar group had significantly higher concentrations of total biogenic amines, when compared to DiarInfl in Co2 (*P* = 0.01) and in Co3 (*P* < 0.05). L-lysine was lowest in the DiarNoInfl group, but putrescine concentration was highest in the DiarNoInfl group. Gender had no effects on total concentration of biogenic amines, while in individual biogenic amines, males showed to have significantly higher levels of putrescine and cadaverine compared to females. Concentrations of L-threonine, agmatine, L-valine and L-lysine were higher in digesta from females compared to males (data not shown).

**Table 2:** Concentration of biogenic amines in digesta (mmol/kg wet sample) from Co2 and Co3

	Groups^1^		
	NoDiar	DiarNoInfl	DiarInfl
Co2			
Biogenic Amines	688 (488–970) ^b^	585 (386–885) ^ab^	416 (316–548) ^a^
L-threonine	43.7 (22.3–85.6)	27.0 (11.3–64.7)	36.6 (22.4–59.8)
Agmatine	43.1 (26.5–70.0)	43.9 (23.4–82.3)	40.6 (28.7–57.5)
DL-methionine	14.7 (2.99–72.7)	12.8 (1.64–100)	14.1 (4.43–45.1)
L-valine	64.1 (38.1–108)	31.1 (15.9–60.8)	53.8 (36.4–79.7)
L-lysine	188 (135–261) ^b^	99.3 (64.8–152) ^a^	146 (115–186) ^ab^
Putrescine	74.4 (48.3–115) ^b^	85.7 (47.7–154) ^b^	27.8 (20.3–38.0) ^a^
Cadaverine	232 (93.7–573) ^b^	211 (70.8–629) ^ab^	86.0 (41.8–177) ^a^
Co3			
Biogenic Amines	631 (443–899) ^b^	689 (452–1050)^b^	450 (342–594) ^a^
L-threonine	32.4 (16.3–64.2)	22.6 (8.9–57.5)	37.7 (22.9–61.9)
Agmatine	45.1 (27.8–73.0)	49.5 (25.8–95.2)	52.2 (36.4–74.7)
DL-methionine	12.6 (2.48–64.2)	11.1 (1.16–105.8)	14.9 (4.55–48.6)
L-valine	53.8 (30.3–95.7)	30.8 (14.6–64.6)	59.1 (39.0–89.7)
L-lysine	175 (125–245) b	94.5 (59.7–150) ^a^	165 (130–211) ^b^
Putrescine	44.7 (29.2–68.3) ^a^	89.4 (51.5–155) ^b^	30.7 (22.5–42.0) ^a^
Cadaverine	219 (89.1–538) ^b^	234 (76.2–717) ^b^	78.0 (38.1–159) ^a^

^1^Diarrheal groups: no diarrheal control (NoDiar; *n* = 10), diarrheal without inflammation in colon (DiarNoInfl; *n* = 6), and diarrheal with inflammation in colon (DiarInfl; *n* = 22). Estimated Marginal Means are reported with their 95% confidence intervals and rows with different superscript letters indicate different EMMs (*P* < 0.05) with pairwise comparison adjusted by BH

Table 3 shows the concentration of NH_4_^+^ and indoles in two segments of colon, Co2 and Co3. Concentration of NH_4_^+^, total indoles and indole-3-methylindole was lowest in the DiarInfl, compared with the NoDiar and DiarNoInfl groups in both Co2 and Co3. Indole-3-acetate was remarkably high in DiarNoInfl for both Co2 and Co3 digesta with 14.1 and 10.7 mmol/kg wet sample, respectively, compared to NoDiar and DiarInfl. Gender and segment had no effects on NH_4_^+^ and indoles (data not shown).

**Table 3:** Concentration of indoles (µg/kg wet sample) and NH_4_^+^ (mmol/kg wet sample) in digesta from Co2 and Co3

	Groups^1^		
	NoDiar	DiarNoInfl	DiarInfl
Co2			
NH_4_^+^	8.45 (5.29–13.5) ^b^	7.82 (4.64–13.2) ^b^	4.76 (3.13–7.25) ^a^
Indoles	41.7 (25.9–67.0) ^b^	37.7 (20.1–70.6) ^b^	17.3 (12.2–24.6) ^a^
Indole-3-acetate	1.31 (0.74–2.33) ^a^	14.1 (6.65–29.8) ^b^	2.27 (1.51–3.4) ^a^
Indole-3-propionate	1.54 (0.93–2.54)	1.11 (0.62–1.99)	1.55 (1.01–2.39)
Indol-1-benzopyrrol	2.98 (1.66–5.35)	2.3 (1.06–5.03)	1.47 (0.95–2.28)
Indole-3-methylindole	22.4 (10.0–50.2) ^b^	9.67 (3.32–28.1) ^ab^	6.78 (3.73–12.3) ^a^
L-Tryptophan	12.5 (4.36–36.1)	10.6 (2.64–42.5)	4.85 (2.23–10.5)
Co3			
NH_4_^+^	11.5 (7.15–18.4) ^b^	7.51 (4.43–12.7) ^ab^	5.82 (3.82–8.86)^a^
Indoles	42.1 (26.1–67.9) ^b^	35.4 (18.8–66.4) ^b^	16.7 (11.9–23.7) ^a^
Indole-3-acetate	1.15 (0.66–2.0) ^a^	10.7 (5.12–22.3) ^b^	1.81 (1.2–2.74) ^a^
Indole-3-propionate	1.35 (0.82–2.23)	0.84 (0.46–1.54)	1.29 (0.84–1.98)
Indol-1-benzopyrrol	3.7 (2.04–6.71) ^b^	1.3 (0.6.0–2.83) ^a^	1.83 (1.2–2.79) ^a^
Indole-3-methylindole	22.3 (9.83–50.6) ^b^	9.0 (3.07–26.4)^ab^	6.46 (3.59–11.6) ^a^
L-Tryptophan	12.8 (4.4–37.1)	13.1 (3.2–53.8)	5.12 (2.37–11.1)

^1^Diarrheal groups: no diarrheal control (NoDiar; *n* = 10), diarrheal without inflammation in colon (DiarNoInfl; *n* = 6), and diarrheal with inflammation in colon (DiarInfl; *n* = 22). Estimated Marginal Means are reported with their 95% confidence intervals and rows with different superscript letters indicate different EMMs (*P* < 0.05) with pairwise comparison adjusted by BH

### Colonic bacterial diversity and composition

#### Alpha diversity

**Fig. 2:**
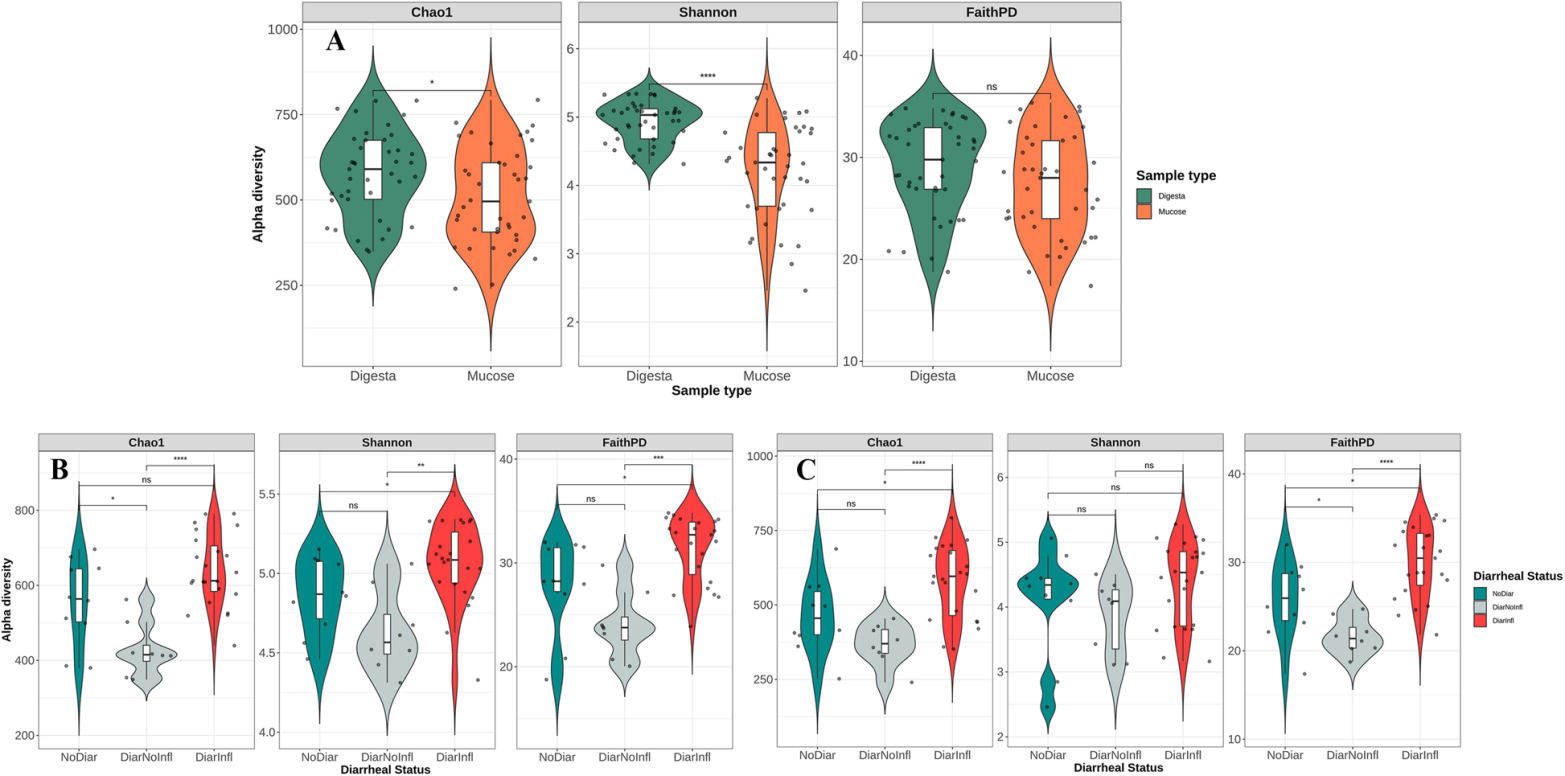
Alpha diversity indices in digesta vs. mucosal samples **A** and separately in digesta **B** and mucosal **C** samples. Alpha diversity for different groups were evaluated by Wilcoxon rank test and differences with *P* < 0.05 were labeled significant

Figure 2A-C show alpha diversity metrics for different samples from digesta vs. mucus (Fig. 2A) and in different diarrheal groups for digesta (Fig. 2B) and mucus (Fig. 2C). Except for FaithPD, all alpha diversity metrics showed to be different between digesta (*n* = 42) and mucus (*n* = 41); digesta samples showed higher Chao1 and Shannon alpha diversity indices compared to mucus. In digesta, DiarNoInfl showed the lowest values (*P* < 0.05) for Chao1, Shannon and FaithPD. The same pattern was observed for mucosal samples except for Shannon, which was constant for all groups. Gender and segment had no effect on alpha diversity indices in digesta and mucus; therefore, these samples obtained from Co2 and Co3 were considered similar.

#### Beta diversity

**Fig. 3:**
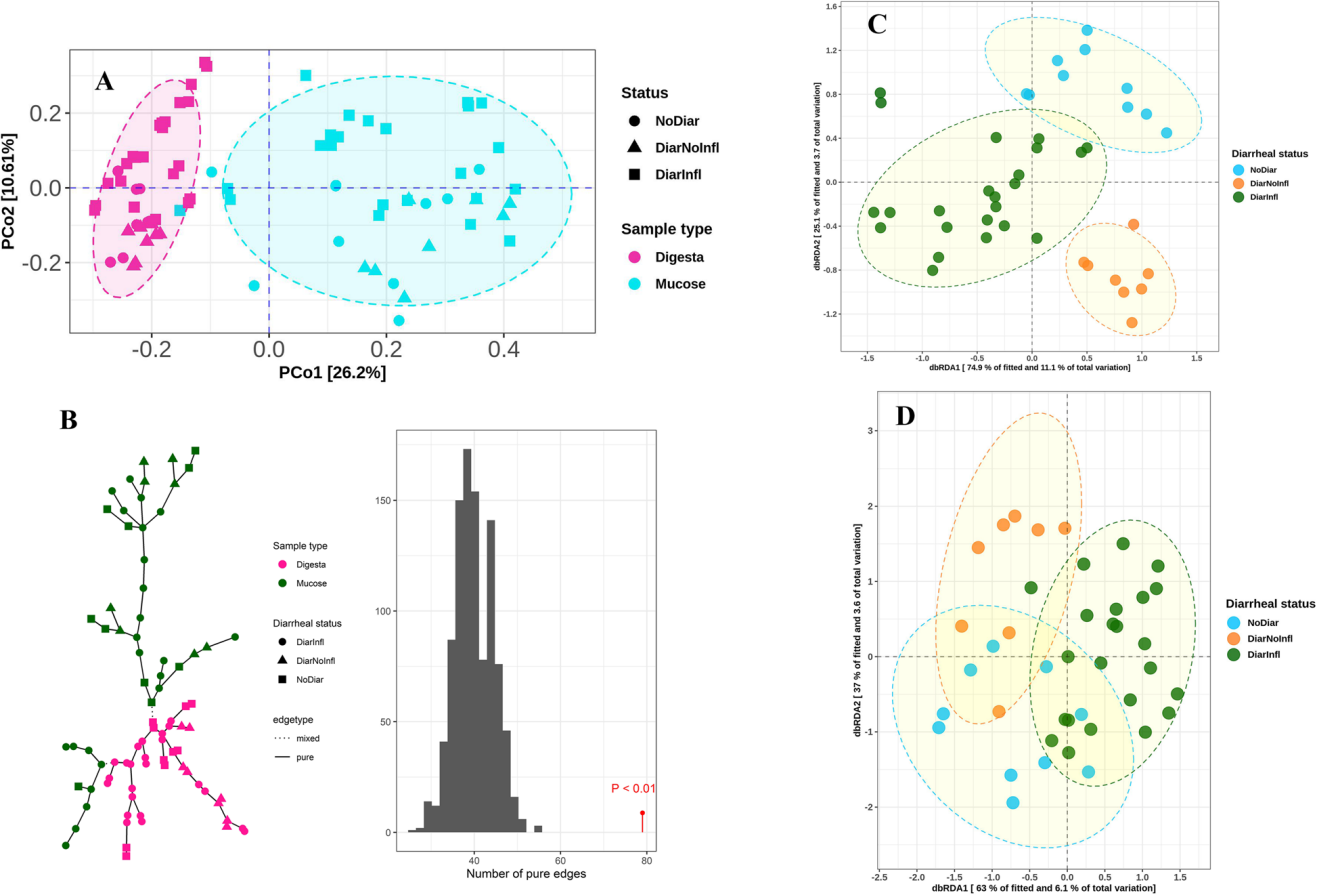
Bi-plot of principal coordinate analysis (PCoA) for log-transformed Bray–Curtis dissimilarity matrix between digesta and mucus A. Variance in Bray–Curtis dissimilarity explained by two most variable axis is presented as percent of total variance. Graph-based analysis of the distributions in bacterial composition in mucus vs. digesta B. Color of nodes represents sample type, and their shapes stand for diarrheal status. The graph is based on Bray–Curtis dissimilarity matrix with maximum distance of 0.35. The histogram of permutation test based on minimum spanning tree (MST) is presented. Ordination plots of samples extracted from the fitted dbRDA model for log-transformed Bray–Curtis dissimilarity matrix in digesta (C; R2 = 0.15, *P* < 0.01) and mucosal (D; R2 = 0.10, *P* = 0.02). The numbers on dbRDA axis for plot C and D represent the proportion of the variation in the fitted data explained by that given axis and it is higher than that relative to the total variation

Differences in bacterial composition between digesta and mucus, and between different groups are shown in Fig. 2. Regardless of diarrheal status, beta diversity based on Bray–Curtis dissimilarity, derived from a Principal Coordinate Analysis (PCoA) showed to be different between digesta and mucus (Fig. 3A), as confirmed by a graph-based analysis (Fig. 3B). The graph shows that samples from digesta formed solid edges together and mixed with mucosal samples, while mucosal samples formed solid edges only together, indicating differences in distribution of data originating from the two sample types (*P* < 0.01). The results of dbRDA showed that in digesta, there was a significant difference (R^2^ = 0.15, *P* < 0.01) between groups based on Bray–Curtis dissimilarity (Fig. 3C) and that the three groups formed separate clusters on ordination plots. In mucus, diarrheal status also had significant influence on beta diversity (Fig. 3D; R^2^ = 0.10, *P* = 0.02). There was no significant difference for beta diversity between Co2 and Co3 in both digesta and mucosal samples. Gender showed no effect on beta diversity for digesta samples; however, gender did influence beta diversity in mucosal samples. To verify the validity of dbRDA model, a test was performed on dispersion of variance around the centroids for sample type and for groups in digesta and mucus separately (Fig. S1A-C). Samples from mucus and digesta were different within group variances (Fig. S1A; *P* < 0.01); therefore, the dataset was split into digesta and mucus, which showed to be variance homoscedastic (*P* > 0.05) according to the group (Fig. S1B-C).

### Bacterial composition and differential abundance

**Fig. 4:**
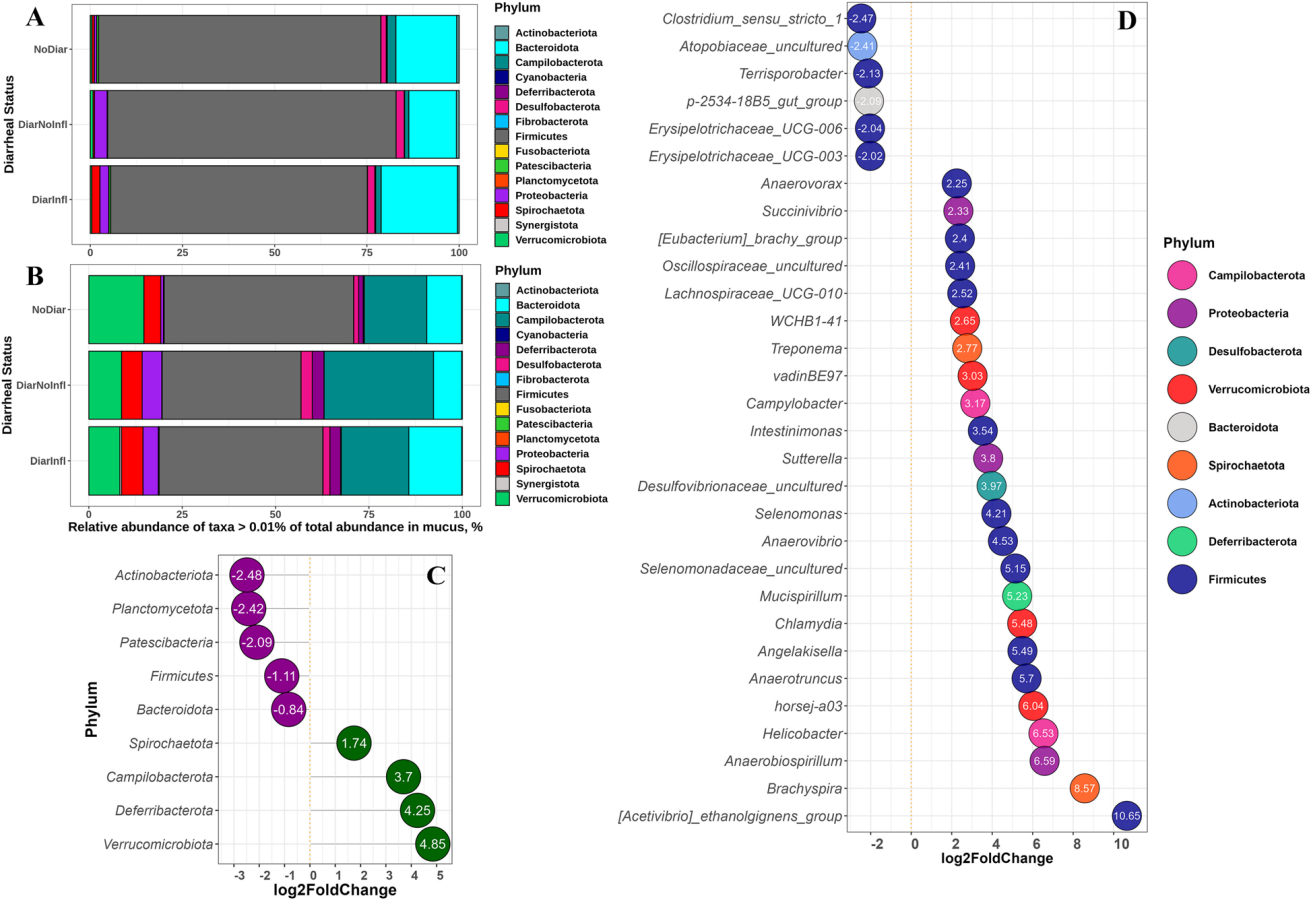
Composition of colonic bacterial phyla in digesta **A** and mucus **B** for different groups, with relative abundance > 0.01% of total abundance. Differential abundance of phyla (FDR < 0.05) in mucosal vs. digesta samples **C**. Differential abundance of genera (FDR < 0.01 and |LFC|> 2) in samples from mucus vs. digesta **D**. Samples for plot C and D are pooled for all diarrheal status and the comparison is between mucosal samples (*n* = 41) vs. samples taken from digesta (*n* = 42). Negative LFC shows lower abundance of taxa in mucus vs. digesta and positive LFC values indicate higher abundance of taxa in mucus compared to digesta

The relative abundances of different phyla were different for digesta compared to mucus and there were also differences between groups (Fig. 4A-B). Numeric relative abundance of *Proteobacteria* was higher for DiarNoInfl and DiarInfl in both digesta and mucus, when compared to the NoDiar group. Regardless of diarrheal status, the relative abundance of *Actinobacteriota*, *Planctomycetota*, *Patescibacteria*, *Firmicutes*, and *Bacteroidota* was higher in digesta compared to mucus, while it was higher for *Verrucumicrobiota*, *Campilobacterota*, *Deferribacterota*, and *Spirochaetota* in mucosal samples vs. digesta samples (Fig. 4C).

At genus level, six genera, four belonging to *Firmicutes*, decreased in abundance when moving from digesta to mucus, while abundance of 24 genera increased in mucus compared to digesta, with the magnitude of this increase being observed for genera *Acetivibrio ethanolgignens* group and *Brachyspira,* 10.7 and 8.57 LFC, respectively (Fig. 4D).

**Fig. 5:**
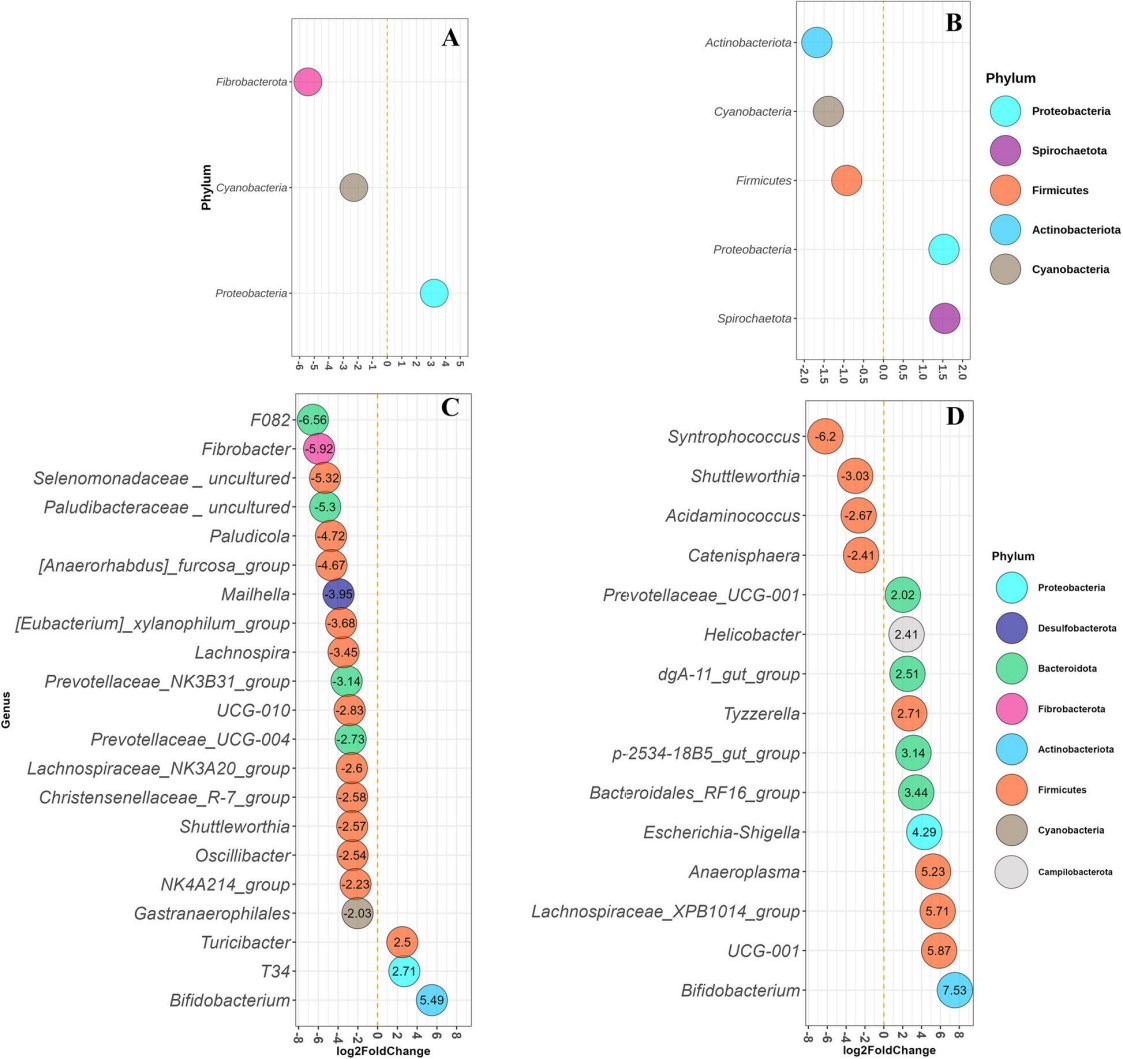
Differential abundance of phyla (FDR < 0.05) in digesta of DiarNoInfl vs. NoDiar **A**, and DiarInfl vs. NoDiar **B**. Differentially abundant genera in digesta of DiarNoInfl vs. NoDiar **C**, and DiarInfl vs. NoDiar **D**. Only genera with FDR ≤ 0.05 and with absolute value of LFC > 2 are presented. Each genus is colored to its representative phylum and labeled with their LFC values

In digesta from DiarNoInfl group, the abundance of *Fibrobacterota* and *Cyanobacteria* phyla decreased, while it increased for *Proteobacteria*, when compared to the NoDiar group (Fig. 5A). The DiarInfl, compared to NoDiar group, showed increased abundance of *Proteobacteria* as well as *Spirochaetota*, while it had reduced LFC for *Actinobacteriota*, *Cyanobacteria* and Firmicutes (Fig. 5B). Comparing DiarInfl with DiarNoInfl group revealed that the former had higher abundance of Spirochaetota (2.0 LFC) and Fibrobacterota and lower in *Proteobacteria* and *Verrucumicrobiota* (Fig. S2A). Digesta from the DiarNoInfl compared to the NoDiar group, showed to have 18 genera reduced in abundance (mainly belong to *Firmicutes*), such as *F082* group, *Fibrobacter*, and *Mailhella*; and three increased in abundance including *Bifidobacterium, T34*, and *Turicibacter* (Fig. 5C). As for DiarInfl, four *Firmicutes* genera were reduced, e.g. *Syntrophococcus* and *Shuttleworthia,* and 11 genera increased in abundance, including *Tyzzerella, Bifidiobacterium*, *Escherichia-Shigella* and *Helicobacter*, when compared to the NoDiar group (Fig. 5D). Comparison of digesta between the two diarrheal groups showed that DiarInfl increased the abundance of 27 genera (chiefly from *Firmicutes* and *Spirochaetota*), compared with DiarNoInlf, and it decreased the abundance of six genera belonging to *Firmicutes* (Fig. S2C).

**Fig. 6:**
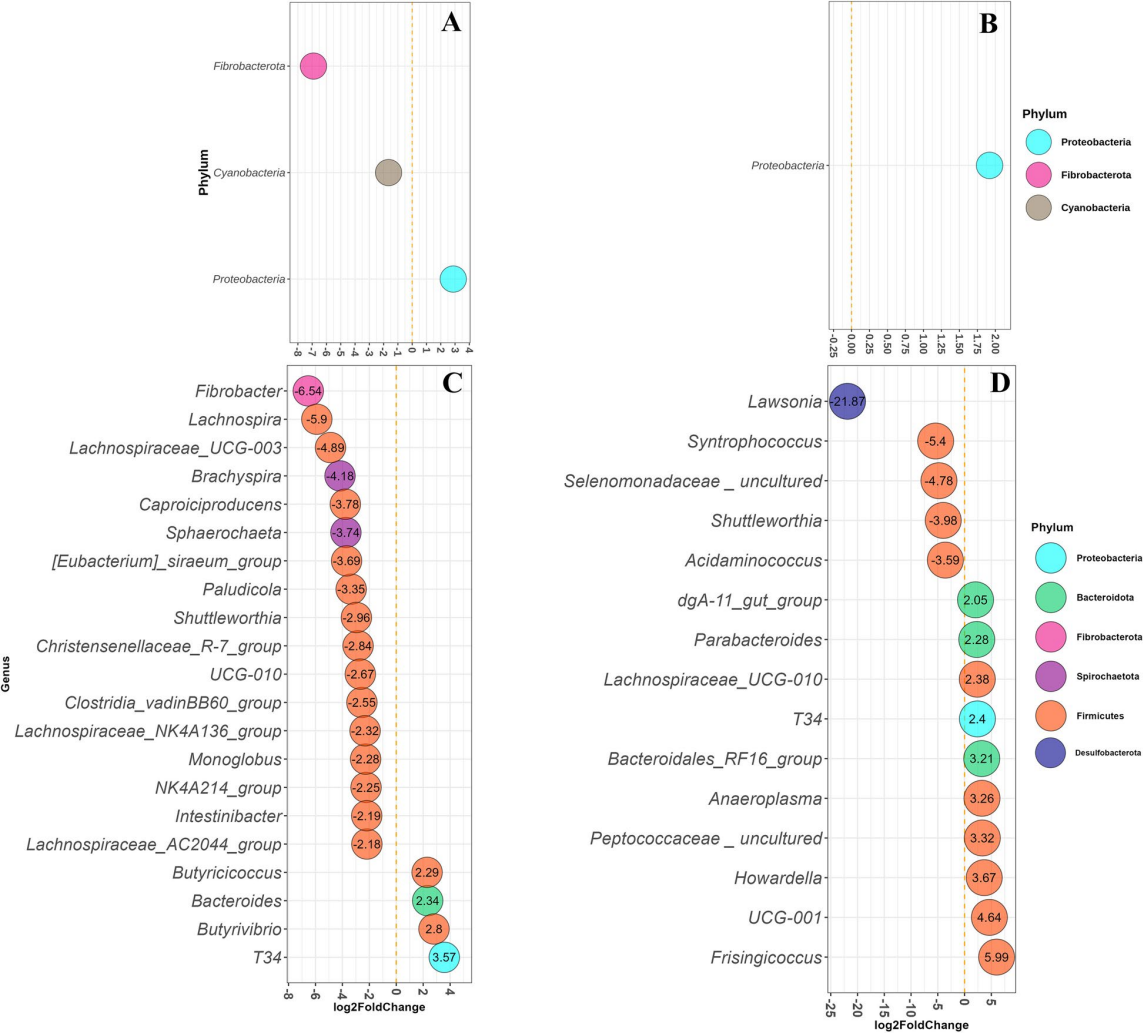
Differential abundance of phyla (FDR < 0.05) in mucus of DiarNoInfl vs. NoDiar **A**, DiarInfl vs. NoDiar **B**. Differentially abundant genera in mucosal samples of pig groups DiarNoInfl vs. NoDiar **C** and DiarInfl vs. NoDiar **D**. Only genera with FDR ≤ 0.05 and with absolute value of LFC > 2 are presented. Each genus is colored to its representative phylum and labeled with their LFC values

In mucus of DiarNoInfl pigs, *Fibrobacterota* (LFC = -7.0) and Cyanobacteria (LFC = -1.50) phyla were reduced and *Proteobacteria* (LFC = 2.90) increased in abundance (*FDR* < 0.05) compared with the NoDiar group (Fig. 6A). DiarInfl vs. NoDiar only resulted in increased abundance of *Proteobacteria* with LFC = 1.80 (Fig. 6B). At the genus level, DiarNoInfl vs. NoDiar showed reduced (FDR < 0.05) abundance of 20 genera, mainly belonging to *Firmicutes*, *Spirochaetota*, and *Fibrobacterota* and increased abundance of four genera in mucus, such as *T34* (Fig. 6C). The DiarInfl group showed to have lower abundance of five genera (e.g., *Lawsonia*, *Syntrophococcus* and *Shuttleworthia*) and higher abundance of 10 mucosal genera, compared with NoDiar (Fig. 6D). In mucosal samples, comparison between DiarInfl and DiarNoInfl showed that DiarInf had lower abundance of *Lawsonia* (from *Desulfobacterota* phylum; Fig. S2B) and higher abundance of *Sphaerochaeta* (belonging to *Spirochaetota*; Fig. S2D).

**Fig. 7:**
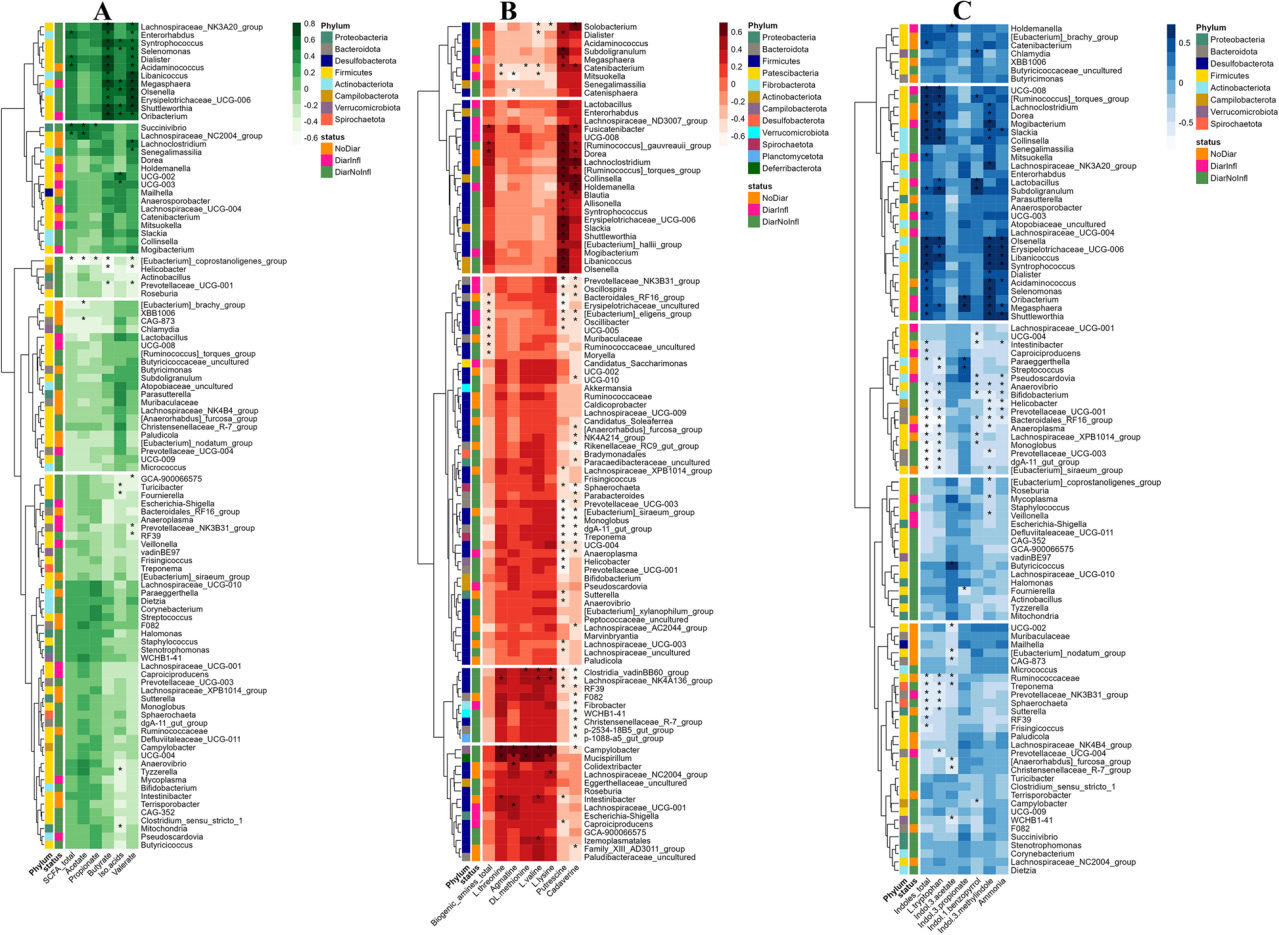
Spearman correlation heatmap of top 100 genera (selected based on their higher variance) and concentration of SCFAs **A**, biogenic amines **B**, and indoles **C** in digesta of pigs in the NoDiar, DiarNoInfl and DiarInfl groups. Significant correlations (FDR < 0.05) are labeled with stars and each genus is colored to its correspondent phylum

Figure 7 represents the association of top 100 genera with different microbial fermentation products in digesta collected from Co2 and Co3 of the three groups. In total, 30, 76, and 74 genera showed significant association with the production of SCFA, biogenic amines, and indoles, respectively. The genera *Shuttleworthia (r* = 0.72 for butyrate; *r* = 0.52 for iso-acid*; r* = 0.72 for valerate), *Syntrophococcus (r* = 0.68 for butyrate; *r* = 48 for iso-acids; *r* = 0.59 for valerate*), Acidaminococcus (r* = 0.71 for butyrate; *r* = 0.60 valerate*), Turicibacter (r* = -0.47 for iso-acid*),* and *Helicobacter (r* = -0.62 for butyrate;* r* = -0.55 for valerate*)* were significantly associated with different SCFA production and they were changed in digesta of DiarNoInfl and DiarInfl vs. NoDiar. In both DiarNoInfl and DiarInfl, *Shuttleworthia* was reduced in abundance compared with NoDiar and this genus was positively associated with butyrate production. In addition, *Syntrophococcus* and *Acidaminococcu* were positively associated with butyrate concentration in digesta, which was reduced in abundance for DiarInfl vs. NoDiar, while *Helicobacter* was increased in abundance, and it showed negative association with butyrate concentration of digesta. *Turicibacter* with negative association with butyrate concentration was increased in abundance in DiarNoInfl vs. NoDiar.

The DiarInfl group showed lower abundance of *Syntrophococcus*, *Acidaminococc,* and *Shuttleworthia* compared with NoDiar and these genera were negatively associated with the concentration of total ammonia, indoles, and indole-3-methylindole.

## Discussion

In total, 30 pigs were initially selected for this survey. Histopathological analysis was the basic premise of the eventual diagnosis of colonic inflammation, but only colonic tissue from 21 pigs were successfully prepared for histology and examined, whereas samples from nine pigs failed in examination. Hence these nine pigs were removed from the dataset. In addition, this study was an observational effort and pigs were assigned to the three diarrheal groups based on the histopathological observations, which resulted in different number of animals in each group. Our results showed that despite absence of specific pathogen shedding in the stool, there were incidences of diarrhea among 8, 11, and 12 week-old pigs. In both diarrheal groups, i.e., with and without inflammation in the colon, there was an approximately two-fold lower dry matter content in feces compared to pigs without diarrhea. This is in line with Pedersen et al. [23], who reported that diarrhea in growing pigs also happens in herds with low pathogen load and that the load of recovered pathogens in the stool does not always correlate with intestinal disease. The results of current study was derived from a small sample size for different diarrheal groups; therefore, they need to be interpreted with caution.

### Microbial fermentation products

The reduced concentration of individual SCFAs such as butyrate and valerate in diarrheal pigs was related to the changes in the composition of DAB in the distal colon. *Shuttleworthia* had a positive correlation with butyrate and valerate production and was reduced in abundance for both DiarNoInfl and DiarInfl groups. Moreover, *Syntrophococcus* and *Acidaminococcus* were positively correlated with butyrate and valerate production, and their abundance reduced in DiarInfl compared with healthy controls. In contrast, *Helicobacter* increased in abundance in DiarInfl vs. NoDiar, demonstrated a negative correlation with digesta butyrate and valerate. Together these observations could indicate the importance of butyrate in gut health. Although digesta pH did not change following the changes in SCFA, it could be expected that increased SCFA concentrations, due to the antimicrobial effects of certain organic acids, can neutralize the virulent effect of pathogens [24] and/or reduce epithelia oxygenation, thereby creating an anaerobic environment [25]. Butyrate metabolism by colonocytes, for instance, is an oxidative reaction, which consumes O_2_ [17] and this can create an epithelial hypoxia (< 1% O_2_) and maintain anaerobic condition [26], oppressing potential facultative anaerobic pathogens. The reduction in butyrate concentration and increased abundance of pathogenic genera was evident in the results of the current study. Digesta butyrate concentration of castrated male pigs was in general higher than in females. Nonetheless, since this study was not designed to investigate the differences between male and female in terms of diarrhea and butyrate concentration in digesta, and due to similar incidence of diarrhea in both genders, we cannot draw a solid conclusion on this gender effect. Total and individual biogenic amine concentrations were lower in digesta of diarrheal groups.

Fermentation of undigested dietary and endogenous proteins results in various metabolites, such as biogenic amines, NH_4_^+^, and indoles [27] and these were lowest in the diarrheal groups. This may be due to the reduced abundance of different genera in the diarrheal groups with putative proteolytic activities. The DiarInfl group showed reduced abundance of *Syntrophococcus*, *Shuttleworthia*, and *Acidaminococcus*, which were positively correlated with the concentration of indoles, NH_4_^+^, and indole-3-methylindole and the reduced concentrations of these compounds was observed in the DiarInfl group. Males, compared with females, showed higher concentrations of putrescine, a biogenic amine involved in mitigating intestinal inflammation through suppressing inflammatory responses in piglets [28].

### Colonic bacterial composition and diversity

The observed differences in alpha and beta diversities between mucus and digesta may reflect the differences in substrate and oxygen availability in the mucus layer compared to luminal digesta [22]. Previous studies also reported a reduced microbiota diversity when moving from luminal digesta into the mucus of piglets [29, 30]. In the mucus layer, the abundance of *Spirochaetota*, *Campilobaceterota*, *Defferibacterota*, and *Verrucomicrobiota* was higher compared with digesta. In mammalian colon, the mucosal surface lining is covered by a mucus layer, mainly composed of heavily glycosylated gel-forming mucins secreted by goblet cells that act as a barrier against pathogens [31]. The carbohydrates in the mucins can be degraded by pig intestinal bacteria to use as an energy source for growth [32]. Here, we demonstrated that in growing pigs, regardless of their diarrheal status, members of *Brachyspira, Chlamydia, Campylobacter,* and *Helicobacter* genus were increased in abundance, when moving from lumen to mucus. The intimate proximity of the MAB and the host, indicates that MAB can be more reliable in evaluating microbial effects on health parameters of the host [21]. Nevertheless, alpha and beta diversity presented a similar pattern for the two diarrheal groups in both mucus and digesta samples. The DiarInfl group showed highest alpha diversity, especially compared to DiarNoInfl, and the dbRDA results indicated a clear separation of distinct clusters on the PCoA plot for these groups. However, the separation was more pronounced for digesta compared to mucus, possibly suggesting a stronger association of bacterial changes in the lumen with the incidence of diarrhea. These changes in diversity could be associated with the incidence of diarrhea in growing pigs, as it was previously suggested that gut microbial dysbiosis was a leading cause of diarrhea in pigs after weaning [33]. Nonetheless, there is a scarce body of literature focusing on how the host-microbe interaction affects the physiology and immunology of pigs [34]; therefore, further studies, especially aimed at understanding the changes in enzymatic pathways of gut microbiota in relation with diarrheal status, are required.

*Mucispirillum* is a Gram-negative genus of the *Deferribacterota* phylum, which showed higher differential abundance (LFC = 5.23) in mucus compared to digesta of distal colon. This was in agreement with Rodríguez-Piñeiro and Johansson [35], who also reported *Mucispirillum* to be highly abundant in the mucosal layer of the distal colon. *Anaerotruncus* is another genus more abundant in mucus, fermenting sugars and proteins of mucin [36], which might explain its higher abundance in mucus.

Composition of DAB and MAB showed differential abundance in the two diarrheal groups compared with the healthy control group. DiarNoInfl pigs had reduced abundance of *Fibrobacterota* and *Cyanobacteria* and increased abundance of *Proteobacteria* in digesta and in mucus. Members of *Fibrobacterota* are involved in cellulose hydrolysis and anaerobic metabolism [37]. Members of *Proteobacteria* are facultative anaerobes, which may explain why they are more abundant in mucus, where oxygen is available [38]. Their increased abundance may be a microbial signature of dysbiosis and it can reflect an unstable structure of the gut microbial community [39]. The direction of changes in colonic digesta and mucosal bacteria in DiarNoInf group was more in an oppressive way, i.e., it was associated with reduced diversity of bacteria and with reduced abundance of two phyla and 18 genera, while increased abundance of one phylum and three genera. However, DiarInfl group, compared with NoDiar, showed to mainly have increased abundance of different genera and decreased only a small number of taxa. *Gastranaerophilales* (*Cyanobacteria*) was reduced in digesta of the DiarNoInfl compared to the NoDiar group. Genera of this order reside in the gut where the environment is basically anaerobic and can also acquire energy via the Embden–Meyerhof pathway that converts simple carbohydrates into pyruvate and through intermediate pathways produces lactate, ethanol, and butyrate [40]. DiarNoInfl also showed reduced abundance of different *Prevotellaceae* groups and *Oscillibacter*, the former being involved in fermentation of plant-based dietary polysaccharides, providing energy for the host [41], and the latter to act as a health-promoting commensal, reducing inflammation in the colon [42]. Compared to NoDiar, the DiarNoInfl showed reduced abundance of 17 genera in the mucus, mainly belonging to *Firmicutes*, *Spirochaetota* and *Fibrobacterota*, and increased abundance of four genera. On the other hand, *Bacteroides* was increased in DiarNoInfl, which is a commensal genus that might be considered to harbor opportunistic pathogens expressing virulence-associated genes when the environment for their adhesion is favorable and they have lower number of substrate competitors [43]. The reduced abundance of *Brachyspira* in DiarNoInfl is in accordance with other studies reporting this genus to be involved in diarrhea with inflammation in growing pigs [3, 4]. This confirms our histology evaluation, as diarrhea in this group was not linked to postmortem lesions of inflammation in the colon. The DiarNoInfl group showed higher abundance of *Butyrivibrio*, which is a butyrate-producing bacteria reported to alleviate symptoms of colitis and diarrhea in mice [44]. Nonetheless, we did not observe significant association of this genus with increased butyrate production in the colon.

Digesta of the DiarInfl pigs showed reduced abundance of four *Firmicutes* genera, including *Syntrophococcus* and *Shuttleworthia*, which are SCFA-producing commensals [45, 46]. Moreover, the DiarInfl group showed a similar pattern in the number of reduced genera in the mucus, as well as a lower abundance of the *Lawsonia* genus in the mucus, when compared to NoDiar and DiarNoInfl. *Lawsonia* is a genus that can infect the distal part of ileum and cause non-inflammatory diarrhea [1, 47]. At phylum level, the digesta of DiarInfl was more dominated by *Proteobacteria* and *Spirochaetota*. The abundance of 10–11 genera increased in digesta and mucus of DiarInfl compared to NoDiar, including *Helicobacter*, *Tyzzerella*, *Escherichia-Shigella*, *Anaeroplasma*, *Bifidiobacterium* and *Frisingicoccus*. *Helicobacter* may develop gastric mucosal ulcers in pigs [48] through increased production of inflammatory cytokines [49], and they could have contributed to colonic inflammation in the DiarInfl group. This confirms previous studies, suggesting that *Campylobacterota* members were involved in growing diarrhea in pigs [50]. It was also previously reported that increased numbers of *Escherichia-Shigella* was seen in the colon of piglets with diarrhea [51] as well as they may be key-players in the development of small intestinal post-weaning diarrhea [52]. *Escherichia-Shigella* are invasive bacteria that can infect the colonic epithelium and cause inflammatory colitis [53]. In addition, increased abundance of *Tyzzerella* in DiarInfl could also be linked with the incidence of inflammation and diarrhea, since overrepresentation of this genus was previously observed in patients with Crohn’s disease [54].

The clinical signs of diarrhea in both DiarInfl and DiarNoInf were identical, while the DiarInfl group showed to be more different from NoDiar in MAB and DAB composition at phylum and genus level compared with the DiarNoInfl group. This may indicate the association of gut microbial changes in the incidence of diarrhea in growing pigs and demands further investigations of their functionality and gene expressions to clarify the etiology of diarrhea with and without colonic inflammation in growing pigs. DiarInfl did not show increased abundance of *Spirochaetota* genera compared with NoDiar, while the group had higher abundance for these genera, when compared with DiarNoInfl. This can possibly indicate that *Spirochaetota* genera co-exist as commensal bacteria and can act as opportunistic pathogens, as genera from *Spirochaetota* are involved in the incidence of growing diarrhea due to colonic inflammation. Furthermore, members of *Spirochaetota* are strict anaerobes that can attach to the mucus layer and degrade mucin to use as a source of energy and their increased in abundance could be an indication of reduced oxygen availability in the mucus layer. In a healthy gut, from digesta to mucus there is a steep gradient of oxygen, with more oxygen being available in the mucus compared to lumen [55]. However, inflammation in the colon causes tremendous changes in metabolic activity, since it is linked to activated neutrophils and monocytes, local proliferation of different cell types, and the activation of multiple O_2_-consuming processes, and with these changes are oxygen-consuming factors that create so-called “inflammatory hypoxia” [56]. In addition, MAB consume oxygen diffused from submucosal tissue, creating extremely low concentrations of oxygen in the intestinal lumen (< 1 mmHg) [55]. Looking at the results from the DiarNoInfl group can give us a putative picture of the directionality of inflammation in the colon in relation to microbial changes. Higher abundance of oxygen-consuming members of  *Actinobacteriota*, *Firmicutes,* and *Proteobacteria* (compared with NoDiar), could have resulted in the exhaustion of oxygen. Reduced abundance of a wide array of different butyrate-producing genera belonged to *Firmicutes* as well as members of strict anaerobic *Spirochaetota* in digesta and mucus of DiarNoInfl compared with NoDiar group, with reciprocate increased abundance of facultative anaerobic *Proteobacteria* could have been indications on somewhat increased available oxygen, suitable for certain pathogens to fester. The reduced butyrate production, possibly consequent to this shift in bacterial composition, may have added up to accumulation of oxygen available in the colon, since butyrate oxidation by colonocytes is an oxygen-consuming process. This can be seen from the higher abundance of some oxygen-tolerant members of *Escherichia-Shigella* (*Proteobacteria*), *Helicobacter (Campilobacterota),* and *Bifidobacterium (Actinobacteriota)*, in the digesta of DiarInfl group compared with control. Moreover, butyrate can have detrimental effects on microbial cells (e.g., pathogens) by reducing pH. On the other extreme, the increase of these pathogens could have somewhat resulted in the infiltration of neutrophils into intercrypts and mucosal layer of colon, which may have resulted in the exhaustion of oxygen. However, Tinevez et al. [57] reported that rather than host neutrophils, members of *Escherichia-Shigella* could deplete mucosa oxygen by aerobic respiration, leading to hypoxic foci of infection. This could be seen in the decreased abundance of *Lawsonia* (Desulfobacterota) and *Proteobacteria* taxa in DiarInfl group compared with NoDiar and DiarNoIfnl, concurrent with increased the number of strict anaerobic *Spirochaetota,* indicating that inflammation in CCD could be related to inflammatory hypoxia caused by oxygen-consuming bacteria. Moreover, the diversity of bacteria in DiarNoInfl was relatively lower compared to DiarInfl, in particular, DiarInfl had higher number of different pathogens. It can, therefore, be speculated that diarrhea in tested pigs occurred due to reduced diversity of microorganisms and butyrate production, possibly due to changes in the diet, and when the state persisted, the resultant accumulation of oxygen could have resulted in propagation of pathogens and diarrhea with inflammation in the colon. In human studies, patients with active UC showed increased abundance of opportunistic pathogens and reduced butyrate-producing bacteria [58]. Nevertheless, to ascertain the validity of such a claim, further investigations through longitudinal analysis are required.

In total, nine phyla and 30 genera were differentially abundant between luminal and mucosal environment; even though, there was a close similarity in the pattern of changes in DAB and MAB for each diarrheal group. For community-based studies, this may indicate that looking into DAB could suffice for investigating the association of colonic microbiota with diarrhea as they, to a great extent, were representative of MAB.

Together, our results show that diarrhea in growing pigs can occur without the presence of specific pathogens, while an underlying strong association was observed between diarrheal status and changes in colonic bacteria. Although the direction of this association is yet to be understood, the changes in the colonic microbial composition were linked to depressed production of SCFA, such as butyrate, in the diarrheal groups. In the DiarNoInfl group, the diarrhea was more associated with the evident reduced diversity and abundance of many bacterial genera, while the DiarInfl group was more associated with increased abundance of different pathogenic genera. This may highlight the importance of SCFA, especially butyrate but perhaps also others in maintaining gut health. It is speculated, that reduced diversity of colonic bacteria in the DiarNoInfl group in combination with reduced butyrate concentration have created a beneficial environment for pathogens that have further induced inflammation. With this speculation, the bacterial composition in the DiarNoInfl group will eventually shift towards the DiarInfl group. However, it demands further longitudinal studies to prove if the DiarNoInfl group is at the onset of developing inflammation in the colonic epithelium.

## Conclusions

Diarrhea in growing pigs was associated with changes in colonic bacterial composition, both for MAB and DAB, as well as in the fermentation patterns. Pigs with diarrhea had lower concentration of butyrate, indoles and biogenic amines. Both MAB and DAB changed in a similar way for groups with diarrhea compared with the healthy control group, indicating their interchangeability for further studies. The DiarNoInfl group showed reduced diversity and abundance of bacteria in both digesta and mucus, while DiarInfl harbored increased numbers of pathogens. With this, we suggest that reduced abundance and diversity of bacteria concurrent with reduced butyrate concentration in the DiarNoInfl group may have paved the way for pathogens and opportunistic pathogens to thrive and induce inflammation in the colonic epithelium, which could further develop into diarrhea with inflammation. However, this allegation needs further longitudinal investigations. For this observational study, a small sample size was allocated to each group, hence the results must be interpreted with caution.

## Methods

### Animals and selection criteria

All animal experimental procedures were carried out in accordance with the Danish Ministry of Justice, Law no. 253/08.03.203 concerning experiments with animals and care of experimental animals and license issued by the Danish Animal Experiments Inspectorate, Ministry of Food, Agriculture and Fisheries, the Danish Veterinary and Food Administration (Approval number: 2018–15-0201–01,470).

In this observational study, we identified and characterized the MAB and DAB of pigs older than 3 weeks post weaning with and without diarrhea. Pigs from 8, 11, and 12 weeks of age were selected from the same herd (Foulum, Aarhus University, Denmark) and they received the same standard weaner diet from weaning on day 28 of age and throughout the study period. All pigs used in this study were donated by the pig research facility at Department of Animal and Veterinary Sciences, Foulum, Aarhus University, Denmark, where the experiment was carried out. The herd had blue specific pathogen free (SPF) health status, did not apply vaccination against *L. intracellularis* and had a minimal use of antibiotics. The pigs selected from the herd to form the present experiment did not receive any antibiotics during the last 3 days before sampling. The pigs were selected from pens after inspection at the day of sampling for clinical signs of CCD, which were loose mucoid stool with dark gray/green color and if pigs showed dirty back/hind area. Overall, a sample size (*n* = 30) of pigs aging were selected, in which 20 showed clinical signs of diarrhea and 10 appeared healthy. In the selection of pigs, gender and weight are randomly distributed and all male pigs are castrated. The selection of in total 30 pigs was performed across pens throughout a 5-week period i.e., each week, three pigs were selected from the randomly selected batch in two rounds (for each round 15 pigs were selected) from two different batches of pigs and on each sampling day, three pigs were euthanized for post-mortem sampling.

### Sampling procedure

On the day of selection, fecal samples were collected by using a rectal swab from the live pig, snap-frozen in liquid nitrogen and stored at -80 °C. For sacrificing pigs, no chemical agents were used and the euthanization was done by a stunt pistol. After sacrificing the pig by stunt pistol followed by bleeding, digesta samples were collected from the mid colon (half-length of entire colon; Co2) and distal colon (last 25% of colon’s length; Co3) without pressing the tissue to avoid mucosal contamination, digesta pH were recorded and the weight of the emptied intestinal segments was registered after all other samples were obtained. For SCFA, indoles and NH_4_^+^, samples of 2.5-5 g digesta were collected in 50-ml tubes with airtight screw caps, placed on ice and then stored at -20 °C until further analysis. The same amount was taken for biogenic amines and collected in separate tubes, placed on ice and stored at -20 °C. Approx. 1 g of the digesta was put in 2-ml vials, snap-frozen and kept at -80 °C for total DNA extraction.

From Co2 and Co3, a 5-cm tissue specimen was isolated, gently emptied (without squeezing) and placed in 10% formalin-containing tubes for histology. Furthermore, 20 cm of colon was sampled immediately after the location where tissue for histology was obtained. The tissue was rinsed thoroughly in three series of sterile 0.9% NaCl solution. By application of a clean objective glass, a thin layer of mucosal scrap was gently obtained to avoid muscular tissue contamination, snap-frozen and stored at -80 °C until analysis by 16S rRNA gene amplicon sequencing for MAB.

### Chemical analysis

Colonic digesta was used for chemical analysis such as SCFA, biogenic amines, indoles, and NH_4_^+^. Quantification of SCFA; acetate, propionate, butyrate, isoacids (isobutyrate and isovalerate), and valerate in digesta samples from Co2 and Co3 were measured by a modification of the capillary gas chromatography method by Richardson et al. [59] as described by Jensen et al. [60], with some modifications by Canibe et al. [61]. Biogenic amines (cadaverine, agmatine, putrescine and tyramine) were quantified by gradient elution on reverse phase HPLC chromatography, as described by Canibe and Jensen [62]. The concentration of indoles in digesta was quantified by gas chromatography according to Jensen and Jensen [63].

### Fecal dry matter and specific pathogen

On the day of euthanizing the pigs, swab fecal samples taken and examined for specific pathogens. *L. intracellularis*, *B. hyodysenteriae,* and *B. pilosicoli* by qPCR according to Stål et al. [64] and since. *L. intracellularis* is a common pathogen infecting ileum and *Brachyspira* spp. are strict anaerobes; we excluded ileal samples from qPCR assays. Fecal dry matter (DM) was quantified by vacuum-freeze drying and the difference between wet and dry samples was considered water and the rest DM.

### Histological analysis

Tissue samples from Co2 and Co3 were fixed in neutral buffered formalin (10% vol/vol) for 24 h and embedded in paraffin. Sections of 5–7 μm were cut and stained with hematoxylin and eosin [65]. Stained sections were evaluated blinded under a light microscope and inflammation was defined as infiltration of inflammatory cells into crypts and/or within lamina propria with or without the presence of edema. Out of 30 selected pigs, histological examination of samples from 9 pigs failed, which resulted in an eventual number of samples from 21 pigs for downstream analysis.

A further classification based on fecal DM content and histology from sampled segments was performed for both healthy and diarrheic pigs to form the eventual groups. Therefore, pigs without clinical signs of diarrhea, with DM content of feces ≥ 18% [66], and no signs of inflammation in the colon were classified NoDiar (*n* = 5), sections from pigs with diarrhea (DM < 18%) but without inflammation as DiarNoInfl (*n* = 4) and sections from pigs with inflammation as DiarInfl (*n* = 12).

### DNA extraction and 16S rRNA gene amplicon sequencing

Total DNA extraction for 16S rRNA gene markers was carried out using approx. 200 mg of digesta and mucosal scrapes from Co2 and Co3. The E.Z.N.A. stool DNA Kit (Omega bio-tek) was used to extract bacterial DNA according to the manufacturer’s instructions. Illuminia’s 16S Metagenomic Sequencing Library Preparation protocol [67], with few modifications as described in Tawakoli et al. [68], was used for the preparation of 16S rRNA gene amplicons. The extracted DNA was amplified in hypervariable regions V3 and V4 of 16S ribosomal RNA gene using primer set Bac 341F (F´:CCTACGGGNGGCWGCAG; with 17 nt) and Bac 805R (R´:GACTACHVGGGTATCTAATCC; with 21 nt) by polymerase chain reaction (PCR). The PCR amplifications were executed on a Veriti® 96-Well Thermal Cycler (Applied Biosystems®) using the following run protocol: Denaturation for 3 min. at 95˚C, cycles for 30 s. each at 95˚C, 55˚C, and 72˚C, and last at 72˚C for 5 min. The final DNA concentration was measured using the Quant-iT HS reagents (Molecular Probes) according to the manufacturer’s instructions. Samples were diluted to approximately 3 ng DNA/µl, pooled and sequenced on a MiSeq desktop sequencer (Illumnia) using 2 × 300 bp chemistry (Illumnia) according to the manufacturer’s instructions.

### Bioinformatics methods for 16S rRNA gene analysis

Raw sequences were quality filtered of the spurious reads, trimmed to remove the forward and reverse primers, and truncated for > 30 Phred score (Q) at minimum of 25% of reads. These steps plus merging and denoising the reads were done by DADA2 package [69] in Quantitative Insights Into Microbial Ecology 2 (Qiime 2) [70] to generate the amplicon sequence variants (ASV) table and representative sequences (repseqs). For denoising, the value for left trim forward, left trim reverse, truncation length forward and truncation length reverse were 17, 21, 280 and 250 nt, respectively. The phylogenetic tree was constructed in Qiime 2 using fragment insertion based on SATé-Enabled Phylogenetic Placement (SEPP) method [71]. For taxonomic classifications, a region-specific classifier based on our primer was created as described earlier by Panah et al. [72].

In the final dataset, only bacterial domain sequences were selected for the downstream analysis. Decontamination of the reads was done based on prevalence of ASVs in the Phylum level in R from which the *SAR324_clade(Marine_group_B)* phylum was identified as contaminant and was removed from the ASV table. Furthermore, ASVs with the prevalence in less than 5 out of 83 samples were filtered out. Relative abundance of different taxa was determined through dividing the number of sequencing reads assigned to different taxa in each sample by the total number of sequencing reads and ASVs below 0.01% abundance of total reads were removed from the count table. Normalized for the same reading depth of 30,000 reads per sample was done by rarefication (sampling without replacement) in phyloseq [73] after which 1 sample and 3 ASVs were removed. After the preprocessing, 82 samples and 869 ASVs passed the filtering and were used for the downstream analysis according to a customized workflow scripted by Panah [74].

### Alpha and beta diversity

Alpha diversity was estimated based on ASV richness (Chao1), Shannon diversity and Faith Phylogenetic diversity (FaithPD) metrics. Chao1 and Shannon were measured from the ASV count table using the phyloseq package and for estimation of FaithPD, the ASV count table and the rooted phylogenetic tree were used as the inputs in *pd* function of picante package [75]. Beta diversity was estimated by Bray–Curtis dissimilarity coefficients, obtained from the *distance* function in phyloseq.

### Analysis of differentially abundant taxa by DESeq2

Normalization of the microbial data and the analysis of differentially abundant taxa have been done by DESeq2 [76] in R at phylum and genus taxonomic levels and all ASVs classified as “uncultured” at family level have been removed for genus agglomeration. Before estimation of the dispersions, the geometric means of the counts in each sample were calculated and used to estimate the effect size of the factors. The results with p-values adjusted for the False Discovery Rate (FDR) by Benjamini-Hochberg (BH) method [77] below 0.05 (FDR < 0.05) were considered for the visualizations based on Log2FoldChange (LFC) in different groups.

### Statistical analysis

The randomization of the herd was done in R statistical package [78] and the main assumption was that the likelihood of occurring diarrhea in growing pigs was equal for pigs aging 8, 11 and 12 weeks; therefore, the samples were considered as the observations of this time spectrum, regardless of the week differences. The relationships between the predictor variables and the expected responses were assessed in R Statistical Package [78]. A Generalized Linear Mixed-Effect Model was used for analysis of the variance for the response variables of chemical data and it was done by *glmer* function in *lme4* package [79]. Estimated marginal means (EMM) of diarrheal status were computed using the *emmeans* package [80] and results are reported with their 95% confidence intervals. The model estimated has the following functional:

$$log(E(Yijkm ))= \alpha + Di + Sj + Gk + Di \cdot Sj + Di \cdot Gk + Sj \cdot Gk + Rm$$

where *Y* is the dependent variable and *α* is the model constant term. The model includes the fixed effects of diarrheal status (*Di*) with three levels (*i* = NoDiar, DiarInf, DiarNoInf), sample type (*S*_*j*_; *j* = digesta and mucus), and gender (*G*_*k*_*; k* = female and male), the second order interaction between fixed effect factors, and the random effect of the rounds of sampling (*R*_*m*_; *m* = r1 and r2). Differences between EMMs have been declared significant at *P* ≤ 0.05. Differences for alpha diversity between groups were evaluated by Wilcoxon rank test. Analysis of variance for beta diversity indices, e.g. Bray–Curtis dissimilarity matrix was done by Distance-based Redundancy Analysis (dbRDA) in R, using *dbrda* function of vegan package [81], with 999 permutations, with block being set to the age factor and the *Condition* parameter set to round and age. The Bray–Curtis dissimilarity matrix was generated on log-transformed ASV counts and before using it for dbRDA model, it was examined for the homogeneity of variance around the centroids of the three diarrheal status, with which it could be concluded that the variances derive from independent variables in the model rather than the dispersion of the observations. The variance dispersion test was done using *betadisper* function in vegan package with the age being set as a constraining block for the permutation (9999 total permutations). Unless otherwise mentioned, corrections for multiple testing were performed by the BH method and FDR < 0.05 was declared significant.

## Supplementary Information

**Additional file 1.**

**Additional file 2.** 

**Additional file 3:** **Table S1.** Number of segments and animals in different variables of the dataset for each group. **Table S2.** Number of samples in each group per analysis. **Fig. S1.** PCo plot of variance dispersion around the centroids for Bray-Curtis dissimilarity in the whole dataset for digesta vs. mucosal samples (**A**), in digesta (**B**), and in mucosal samples (**C**). *P*-value below 0.05 indicates lack of homogeneity of variance around centroids. **Fig. S2.** Differential abundance of phyla (FDR < 0.05) for DiarInfl vs. DiarNoInfl in digesta (**A**) and in mucus (**B**). Differentially abundant genera for DiarInfl vs. DiarNoInfl in digesta (**C**) and in mucus (**D**). Only genera with FDR ≤ 0.05 and with absolute value of log2FoldChange > 2 are presented. Each genus is colored to its representative phylum and labeled with their correspondent log2FoldChange values.

## Data Availability

Amplicon sequences for this study are available at the NCBI Sequence Read Archive (SRA) with accession no. PRJNA914203. Scripts for analysis of data in R can be found in this link and in the Additional file 1: Additional file 1_workflow_R.pdf which was made using knitr package in R [82]. The metadata for the analysis could be accessed via this link and in Additional file 2: Additional file 2_MAB_DAB_metadata.xlsx. Supplementary figures are available in Additional file 3: Additional file 3_Supplementary materials_MAB_DAB_manuscript_25-04–2023.docx.

## Abbreviations

CCD: Colitis-complex diarrhea
DAB: Digesta-associated bacteria
MAB: Mucus-associated bacteria
NoDiar: Healthy no diarrheal
DiarNoInfl: Diarrheal without inflammation in colon
DiarInfl: Diarrheal with inflammation in colon
SCFA: Short-chain fatty acids
SPF: Specific pathogen free
DM: Dry matter
PCR: Polymerase chain reaction
ASV: Amplicon sequence variants
SEPP: SATé-Enabled Phylogenetic Placement
FaithPD: Faith Phylogenetic Diversity
EMM: Estimated marginal mean
dbRDA: Distance-based redundancy analysis.
LFC: Log2FoldChange
