# Supplementary material for: Composition of mucus- and digesta-associated bacteria in growing pigs with and without diarrhea differed according to the presence of colonic inflammation
Source: BMC Microbiol. 2023 May 20;23:145. doi: 10.1186/s12866-023-02874-1 (PMC10199627; doi:10.1186/s12866-023-02874-1)
Supplement: Supplementary file 1 — Additional file 1. [file 12866_2023_2874_MOESM1_ESM.pdf]

16S rRNA amplicon analysis

Farhad M. Panah

2022-01-09

In this workflow the main steps for 16S rRNA amplicon data analysis are presented. This sheet is related to the manuscript titled "Composition of mucus- and digesta-associated bacteria in growing pigs with and without diarrhea differed according to the presence of colonic inflammation".

1. Loading library

```
library(tidyverse)
library(phyloseq)
library(DESeq2)
library(ampvis2)
library(qiime2R)
library("knitr")
library("gridExtra")
library("BiocManager")
library("DECIPHER")
library("phangorn")
library("vegan")
library("ggrepel")
library("devtools")
library("reshape2")
library("ggnetwork")
library("intergraph")
library("biomformat")
library("pheatmap")
library("glue")
```

2. Importing artifacts from qiime2 to r

```
#making phyloseq objects from qiime files
ps <- qiime2R::qza_to_phyloseq(features = "~/data/ccd/table-ccd.qza",
                       taxonomy = "~/data/ccd/taxonomy-ccd.qza",
                     tree = "./tree-ccd.qza")
```

```
repseqs <- qiime2R::read_qza("~/data/ccd/repseqa.qza")$data
#we need to merge the refseqs like this since in above fun, there is no argument for that
ps= merge_phyloseq(ps, repseqs)

# Metadata
#importing the metadata
metadata <- read.table("./metadata-ccd.tsv", header = TRUE, sep = "\t")

#modifying metadata
metadata$pig_no <- paste("pig", sep = "", metadata$pig_no)
metadata$sample.id = gsub("-", ".", metadata$sample.id)#instead of the dash, we put ".",
in the sample names
rownames(metadata) <- NULL
metadata <- column_to_rownames(.data = metadata, var = "sample.id")
metadata$pen <- paste("pen", sep= "", metadata$pen)
metadata$round <- ifelse(metadata$pig_no %in% c("pig1","pig2", "pig3", "pig4",
"pig5", "pig6","pig7", "pig8", "pig9", "pig10", "pig11", "pig12", "pig13", "pig14",
"pig15"), "r1", "r2") %>%
  factor(levels = c("r1", "r2"))
metadata$age <- factor(paste0("w", metadata$age), levels = c("w8", "w11", "w12"))
metadata$sample_type <- ifelse(metadata$sample_type == "digesta", "Digesta", "Mucose")

metadata = metadata %>% dplyr::select(1:5, 55, 6:54)
col.nam <- c('pig_no','age','sex','weight','pen','round','sample_type','Segment',
'pH','diar_qual','diar_quant','DM','diar_score','neut_infilt','inflammed',
'status','SCFA','Acetate','Propionate','Butyrate','Iso.acids','Valerate',
'Indoles','L.tryptophan','Indol.3.acetate','Indol.3.propionate','Indol.1.benzopyrrol',
'Indol.3.methylindole', 'Biogenic_amines','L.threonine','Agmatine','DL.methionine',
'L.valine','L.lysine','Putrescine','Cadaverine','Ammonia')



metadata = metadata[,colnames(metadata) %in% col.nam]
colnames(metadata )[8] <- "segment"

metadata = metadata %>% mutate(stat = ifelse(status == "Diar.Infl", "DiarInfl",
ifelse(status == "Diar.NoInfl", "DiarNoInfl", "NoDiar")))

#converting non numeric and non-logical variables to factors
for(i in seq_len(ncol(metadata))) {
 if(!is.numeric(metadata[[i]]) && !is.logical(metadata[[i]]) &&
 !is.integer(metadata[[i]])) {
    metadata[[i]] = as.factor(metadata[[i]])  } else {
     metadata[[i]]
 }
}

metadata$status <- factor(metadata$status, labels = c("NoDiar", "Diar.NoInfl",
"DiarInfl"))#reordering the status factors
metadata$stat <- NULL

#changing sample names
asvs <- otu_table(ps) %>% as.matrix
```

```
colnames(asvs) <- gsub("-", ".", colnames(asvs))

pst = phyloseq(otu_table(asvs, taxa_are_rows = TRUE), phy_tree(ps),
sample_data(metadata), refseq(ps), tax_table(ps))
```

3. Filtering and preprocessing of reads

```
#removing unassigned and NA phylum taxa
pst <- subset_taxa(pst, !is.na(Phylum) & !Phylum %in% c("", "uncharacterized",
"unassigned"))

#keeping only bacterial kingdom
pst <- subset_taxa(pst, Kingdom %in% "d__Bacteria")

#Removing domain annotation from kingdom bacteria (d__Bacteria)
temp.taxa <- tax_table(pst) %>% as.data.frame

kings <- apply(dplyr::select(temp.taxa, 1), 2, function(x)  substr(x, start = 4, stop =
nchar(x)))
temp.taxa$Kingdom<- kings[rownames(kings) %in% rownames(kings),]
tax_table(pst) <- tax_table(as.matrix(temp.taxa))
```

Taxonomic filtering based on prevalence: supervsed

```
#monitoring the number of the samples in which the prevalence of a taxon is at least one
prevdf <- apply(otu_table(pst),ifelse(taxa_are_rows(pst), 1, 2), function(x){sum(x>0)})
prevdf <-data.frame(ASVprev = prevdf,
                   TaxaAbund = taxa_sums(pst),
                   tax_table(pst))
head(prevdf)
#Find out the phyla that are of mostly low-prevalence features by computing the total and
average prev of features in Phylum
plyr::ddply(prevdf, "Phylum", function(df){cbind(means = round(mean(df$ASVprev), 2), sums
= round(sum(df$ASVprev),2))}) %>% mutate(sanity = ifelse(means == sums, "TRUE", "FALSE"))
#the results show that the mean and the prevalence of SAR324_clade(Marine_group_B), a
marine bacteria, is the same indicating it's only once occurance in the dataset. and it
will be removed
#the same is true about WPS-2, although it has been detected in the pig gut based on the
prevoius studies.
pst = subset_taxa(pst, !Phylum %in% "SAR324_clade(Marine_group_B)")

# A backup of pst file for differential abundance analysis and for richness alpha
 pst.res <-pst
```

Filtering ASVs based on their prevalence threshold of occurence in n samples across all samples

```
# This means that each ASV should have appeared at least in n samples to be kept.
asv.filter = function(asvtab, n.samples = 1 ){
  filter.threshold <- n.samples/ncol(asvtab) *100 # In how many samples out of total
samples an ASV should have occured
  table_count <- apply(otu_table(asvtab), 2, function(x) ifelse(x>0, 1, 0)) %>%
as.data.frame()
  suspected_ASV = table_count[which((rowSums(table_count)/ncol(table_count))*100 <
filter.threshold),] %>% rownames()

  return(suspected_ASV)
}

(sus_ASV = asv.filter(asvtab = otu_table(pst), n.samples = 2) ) %>% length

pst = subset_taxa(pst, !taxa_names(pst) %in% sus_ASV)


#or you could also do it by the phyloseq function

condition <- function(x) x>0
TaxaTokeep <- genefilter_sample(pst,condition,2)
#pst = subset_taxa(pst, taxa_names(pst) %in% TaxaTokeep)
```

Removing singletones based on abundance

```
#A function to find singletones. You need to be careful about this step!
out.ASV = function(phyloseq, threshold =1, binwidth = 0.01) {

#Loading necessary pkgs
  pacman::p_load(glue, tidyverse, reshape2, ggrepel, S4Vectors) # nolint
#This function requires phyloseq, tidyverse and glue packages to be loaded.
    if (sum(colSums(otu_table(phyloseq)))/ncol(otu_table(phyloseq)) == 100 ) {#making the
    relative abundance table
                    rel_abund = as(t(otu_table(phyloseq)), "matrix")
    } else if (sum(colSums(otu_table(phyloseq)))/ncol(otu_table(phyloseq)) == 1) {
                    rel_abund = as(t(otu_table(phyloseq)), "matrix")
                    } else {
                    rel_abund = as(t(apply(otu_table(phyloseq),
                    ifelse(taxa_are_rows(phyloseq), 1,2),
                    function(x) x/sum(x))), "matrix")
                    }


      names.single = apply(rel_abund, 1, function(x){ifelse(x == threshold, TRUE,
                                                ifelse(x == sum(x), TRUE, FALSE))})
                                                %>%
        reshape2::melt() %>% filter(value == TRUE) %>% dplyr::select(2) %>% pull%>%
as.vector()
```

```
    if (length(names.single) == 0 ) {
          print(glue("WOW! {length(names.single)} singletones detected in this dataset"))
                 qplot.noSing = qplot(rel_abund, geom = "histogram", binwidth = binwidth,

                  show.legend = F, main = "Frequency count of relative abundance, no
                  singletones detected") +
         xlab ("Relative abundance in samples") +
                   ylab("Frequency") + theme_bw()

    return(structure(list(qplot.noSing)))

                        } else {

single.ASV = rel_abund[rownames(rel_abund) %in% names.single,]
single.ASV[single.ASV == 0] <- NA # A separate dataset for annotation of singletones on
the barplot

         qplot.withSing = qplot(rel_abund, geom = "histogram", binwidth = binwidth,
        main = "Frequency count of relative abundance with singletones") +
        geom_bar(aes(single.ASV), fill = "red",  color = NA, width = binwidth)+
                       xlab ("Relative abundance in samples") + ylab("Frequency") +
                       geom_label_repel(aes(x = 1, y =length(rel_abund)/5),
                       label.padding =  unit(0.55, "lines"),
                       label = glue("{length(names.single)}\n Singletones"), color =
                       "black") +
           theme_bw()

                       qplot.rmSing = qplot(rel_abund[!rownames(rel_abund) %in%
names.single, ], geom = "histogram",
                       binwidth = binwidth, main = "Frequency count of relative abundance
                       without singletones") +
                       xlab ("Relative abundance in samples") + ylab("Frequency")+
                       theme_bw()

                       print(glue('Oh no..! {length(names.single)} singletones detected
                       in the dataset'))
                       return(structure(list(qplot.withSing, qplot.rmSing,
                       unlist(names.single))) )

                        }


        }

single.test = out.ASV(phyloseq = pst, threshold = 1, binwidth = 0.1)
singletones = single.test[[3]] #here you can extract the names of the singletones

single.test[[1]]#to show the plot with singletones
single.test[[2]]#to show the plot without singletones

#Now you can remove the singletones from your pst file as follows:
pst = subset_taxa(pst, !taxa_names(ps)%in% singletones)
```

```
rm(single.test)
```

4. Alpha diversity

4.1. Rarefaction

```
library(MicrobiotaProcess)


#This takes a bit of time
ps_rar_curve <- MicrobiotaProcess::ggrarecurve(obj = pst,
                    indexNames = c("Observe", "Shannon"),
                   chunks=400,
                    theme(legend.spacing.y = unit(0.02, "cm"),
                          legend.text = element_text(size = 6)), show.legend=F)



ps_rar_curve + theme_bw() + geom_vline(xintercept = 20000, lty = 2, color = alpha("red",
0.5)) +
  ggtitle("Rarefaction curves each line is a sample")

ggsave("./Alpha/rarefaction.curve.jpeg", device = "png", dpi = 300, height = 6, width =
9)

#rarefying the table with minimum depth of 30000 reads per sample
ps_rar = rarefy_even_depth(pst, sample.size = 30000, replace = FALSE)#in this depth we
have lost one sapmle (F.29) and no ASVs.

# Taxonomic filtering based on abundance for rarefied data: supervused
## Abumdance: ASV > 0.0001% overall abundance across all samples
total.depth <- sum(otu_table(ps_rar))
totAbuThreshold <- 1e-4 * total.depth
ps_rar <- prune_taxa(taxa_sums(ps_rar)>totAbuThreshold, ps_rar)

ps_rar
```

4.2. Alpha diversity metrix

```
#Calculating the alpha diversity indexes
Chao1 = estimate_richness(pst.res, split = TRUE, measures = "Chao1")#for richness, we
don't use rarefied table

#Shannon
Shannon = estimate_richness(ps_rar, split = TRUE, measures = "Shannon")
```

```
#Faith Phylogenetic Diversity
library(picante)
FaithPD = picante::pd(samp = t(otu_table(ps_rar)), tree = phy_tree(ps_rar))$PD

#adding the indexes to the metadatas
sample_data(ps_rar) <- data.frame(sample_data(ps_rar),
Chao1=Chao1[!rownames(Chao1)%in%"F.29",][[1]], Shannon = Shannon$Shannon, FaithPD =
FaithPD) #note that we have removed that sample which has been removed by rarefaction.
```

4.3.Visualizing alpha diversity index

```
library(ggpubr)
library(reshape2)

alpha.ccd = sample_data(ps_rar) [ , c(1:16, 38:40)]  %>% data.frame

long_mtdat <- melt(alpha.ccd)
long_mtdat<- long_mtdat[long_mtdat$variable %in% c("Chao1", "Shannon", "FaithPD"),]



long_mtdat$variable <- factor(long_mtdat$variable , levels =  c("Chao1", "Shannon",
"FaithPD"))


#comparison between mucusal and digesta

alpha_ccd = ggplot(long_mtdat, aes(x = sample_type, y = value)) +
  geom_violin(aes(fill = sample_type), trim = F) +
  stat_compare_means(paired = FALSE, comparison = list(c("Digesta", "Mucose")), method =
  "t.test", label = "p.signif") +
  geom_boxplot(width = 0.15) + geom_jitter(color = "black", alpha = 0.5)+
  facet_wrap(~long_mtdat$variable, scales = "free_y") +
  theme_bw() +
  scale_fill_manual(values = c( "aquamarine4", "coral")) +
  theme(legend.title = element_text( size = 15, face = "bold"),
  axis.title.x = element_text( face = "bold", size = 15), axis.text.x = element_text(
  size = 15),
  axis.title.y = element_text( face = "bold", size = 15),
  axis.text.y = element_text( size = 15), strip.text.x = element_text( size = 15, face =
  "bold")) +
  labs(fill = "Sample type",  y = "Alpha diversity",
  title = "Alpha diversity metrics for mucosal vs. digesta samples.\n Means are compared
  by unpaired t.test") +
  xlab("Sample type")

alpha_ccd
ggsave(plot = alpha_ccd, "./Alpha/alpha_ccd_digest_mucose.jpeg", device = "jpeg", width =
15, dpi =300)
rm(alpha_ccd)
# digesta based on dirrheal status
```

```r
alpha_ccd_digest = ggplot(long_mtdat[long_mtdat$sample_type == "Digesta",], aes(x =
status, y = value)) +
  geom_violin(aes(fill = status), inherit.aes = T, trim = FALSE) +
  geom_boxplot(fill = "white", position = position_dodge(0.9), width = 0.1, outlier.shape
  = NA) +
  geom_jitter(color = "black", alpha = 0.5)+
  facet_wrap(~long_mtdat[long_mtdat$sample_type == "Digesta", "variable"], scales =
  "free_y") +
  scale_fill_manual(values = c("cyan4", "azure3", "brown1"))+
  labs(fill= "Sample type", y = "Alpha diversity",
  title = "Alpha diversity metrics for digesta samples,\n the means are compared by
  unpaired t.test", y= "Values",
  x = "Sample type") + theme_bw() + labs(fill = "Diarrheal Status", x = "Diarrheal
  Status") +
  theme(legend.title = element_text( size = 15, face = "bold"), axis.title.x =
  element_text( face = "bold", size = 15), axis.text.x = element_text( size = 15),
        axis.title.y = element_text( face = "bold", size = 15),
        axis.text.y = element_text( size = 15), strip.text.x = element_text( size = 15,
        face = "bold"))# + coord_flip()

#adding significance signes
my.compare = list(c("DiarNoInfl", "NoDiar"), c("DiarInfl", "NoDiar"), c("DiarInfl",
"DiarNoInfl"))

alpha_ccd_digest = alpha_ccd_digest + stat_compare_means(paired = F, comparisons =
my.compare, method = "t.test", label = "p.signif")

alpha_ccd_digest

ggsave(filename =  "./Alpha/alpha.ccd.digesta.jpeg", plot = alpha_ccd_digest, device =
"jpeg", width = 15, dpi = 300)
rm(alpha_ccd_digest)


#mucusal samples
alpha_ccd_mucose = ggplot(long_mtdat[long_mtdat$sample_type == "Mucose",],
                          aes(x = status, y = value)) +
  geom_violin(aes(fill = status), inherit.aes = T, trim = FALSE) +
  geom_boxplot(fill = "white", position = position_dodge(0.9),
               width = 0.1, outlier.shape = NA) +
  geom_jitter(color = "black", alpha = 0.5)+
  facet_wrap(~long_mtdat[long_mtdat$sample_type == "Mucose","variable"], scales =
  "free_y") +
  scale_fill_manual(values = c("cyan4", "azure3", "brown1"))+
  labs(fill= "Diarrheal Status",  y = "Alpha diversity",
       title = "Alpha diversity metrics for mucosal samples,\n the means are compared by
       unpaired t.test",
       y= "Values",
  x = "Diarrheal Status")+ theme_bw() +
  theme(legend.title = element_text( size = 15, face = "bold"),
        axis.title.x = element_text( face = "bold", size = 15),
        axis.text.x = element_text( size = 15),
        axis.title.y = element_text( face = "bold", size = 15),
```

```
       axis.text.y = element_text( size = 15),
       strip.text.x = element_text( size = 15, face = "bold")) +
  stat_compare_means(paired = F, comparisons = my.compare, method = "t.test",label =
  "p.signif")



ggsave(filename =  "./Alpha/alpha.ccd.mucose.jpeg", plot = alpha_ccd_mucose, device =
"jpeg", width = 15, dpi = 300)
rm(alpha_ccd_mucose)
```

5. Beta diversity

5.1. Ordination plots

```
ps.log <- transform_sample_counts(ps_rar, function(x) log(1+x))#then we log transform
them to account for the zero inflation. Here we literaly add a psodocount to the zero
counts

#bray: digesta vs. mucus using rarefied data.

#bray PCoA
bray.pcoa=ordinate(ps.log, method="PCoA", distance = "bray")
evals<-bray.pcoa$values$Eigenvalues

bray.pcoa.plot = plot_ordination(ps.log, bray.pcoa, color="sample_type",
                                 shape = "status", title = "Bray-Curtis PCoA plot,
                                 digesta vs. mucose. LogT")+
  labs(col="Sample type", shape = "Status")+ geom_point(size = 5) +
   coord_fixed(sqrt(evals[2]/evals[1])) +
  stat_ellipse(aes(group = sample_type, fill = sample_type),
   show.legend = F, type = "t", level = 0.9, lty = 2, geom = "polygon", alpha = 0.1) +
    labs(x = sprintf("PCo1 [%s%%]", round(evals/sum(evals)*100,1)[1]),
      y = sprintf("PCo2 [%s%%]", round(evals/sum(evals)*100, 2)[2]))+
       scale_color_manual(values = c("maroon2",  "turquoise2"))+
         scale_fill_manual(values = c("maroon2",  "turquoise2")) +
            geom_vline(xintercept = 0, lty = 2, alpha = 0.5, color = "blue") +
             geom_hline(yintercept = 0, lty = 2, alpha = 0.5, color = "blue") +
             theme_bw()+
              theme(axis.title = element_text(face = "bold"),
                legend.title = element_text(size = 10, face = "bold"),
                 legend.text = element_text(face = "bold"),
                   axis.text = element_text(size = 15))


ggsave("./Beta/bray.pcoa.dig.vs.muc.jpeg", dpi = 300)
rm(bray.pcoa.plot)
```

Gloomer: A funciton that wraps 'tax_glom()' funciton and adds neat unique names to the taxa

```
# Gloomer

#A function to create unique names for each ASV. If species is set as the taxa level, it
removes any NA in Order level then attempts to use the name of one level higher taxa for
those who have similar names, e.g. uncultured_bacterium

gloomer = function(ps = data, taxa_level = taxa_level, NArm = "TRUE"){
    rank.names = c('Kingdom','Phylum', 'Class', 'Order', 'Family', 'Genus', 'Species')


#====================Sometimes in genus level, we might have multiple uncultured
organisms, which if we want to make unique out of them for the species level it won't
work====
    #since adding uncultured to uncultered is sill duplication. therefore if the
    taxa_level is set to species we first make a unique genus and then we go further to
    the speices===#

#Removing unculured Family
ps = subset_taxa(ps, !Family %in% c("uncultured", "NA", "uncategorized", "unassigend",
"", " "))

if(taxa_level == "Species") {

    ps = subset_taxa(ps, !Genus %in% NA)#we remove genus tagged NA
tax_table(ps)[, taxa_level] <- ifelse(is.na(tax_table(ps)[, taxa_level]),
paste0("unknown"), paste(tax_table(ps)[, taxa_level]))#convert NA in species into unknown

  physeq = tax_glom(physeq = ps, taxrank = taxa_level, NArm = NArm)
  taxdat = tax_table(physeq)[, seq_along(rank.names[1:which(rank.names == taxa_level)])]

   taxdat = taxdat[complete.cases(taxdat),] %>% as.data.frame
    otudat = otu_table(physeq)

#first take care of the uncultured genus
taxdat[,6] = ifelse(taxdat[,6] %in% c("uncategorized", NA, "uncultured", "unassigend",
"", " "),
       paste0("[", taxdat[,length(rank.names[1:which(rank.names=="Genus")])-1], "]", "_",
       taxdat[,6]), taxdat[,6])

spec1 = taxdat[, taxa_level] %>% as.vector
spec2  = taxdat[, taxa_level] %>% as.vector

    uni  = matrix(NA, ncol = length(spec2), nrow = length(spec1))
    for(i in seq_along(spec1)){
        for(j in seq_along(spec2)){
    uni[i, j] = ifelse(spec1[i] == spec2[j] , "TRUE", "FALSE")
    }
        }

rownames(uni) <-spec1
```

```
colnames(uni) <- spec2
uni[upper.tri(uni, diag = TRUE)] = 0 #get rid of diagonals and upper triangle

duplis = uni %>% reshape2::melt() %>% filter(value == "TRUE")

if(dim(duplis)[[1]] > 0) {
duplis = uni %>% reshape2::melt() %>% filter(value == "TRUE") %>% dplyr::select(1) %>%
unique() %>% unlist %>% as.vector
taxdat = taxdat %>% mutate( uni= ifelse(taxdat[, taxa_level] %in% duplis,
                    paste0("[",
                    taxdat[,length(rank.names[1:which(rank.names==taxa_level)])-1], "]",
                    "_", taxdat[,taxa_level]), taxdat[,taxa_level]))

#check if all the names are unique at species level, otherwise we will bring family
instead of genus
   dupies <-  taxdat[duplicated(taxdat[,"uni"]), "uni"]
    if(length(dupies)>0) {
        taxdat = taxdat %>% data.frame %>% mutate( uni2= ifelse(taxdat[, "uni"] %in%
dupies,
                    paste0("[",
                    taxdat[,length(rank.names[1:which(rank.names==taxa_level)])-2], "]",
                    "_", taxdat[,"uni"]), taxdat[,"uni"]))

        taxdat[, taxa_level] = taxdat[, "uni2"]
        taxdat[, "uni"] <- NULL
        taxdat[, "uni2"] <- NULL
        taxdat <- as(taxdat, "matrix")
        rownames(otudat) <- taxdat[rownames(taxdat) %in% rownames(otudat), taxa_level]
        rownames(taxdat) <- taxdat[, taxa_level]
        taxdat <- tax_table(taxdat)
        taxa_names(physeq) <- taxa_names(taxdat)
        tax_table(physeq) <- taxdat
        otu_table(physeq) <- otudat

    }
    else
    {

taxdat[, taxa_level] = taxdat[, "uni"]
taxdat[, "uni"] <- NULL
taxdat <- as(taxdat, "matrix")
rownames(otudat) <- taxdat[rownames(taxdat) %in% rownames(otudat), taxa_level]
rownames(taxdat) <- taxdat[, taxa_level]
taxdat <- tax_table(taxdat)
taxa_names(physeq) <- taxa_names(taxdat)
tax_table(physeq) <- taxdat
otu_table(physeq) <- otudat
           }

} else {

taxdat <- as.matrix(taxdat)
taxdat <- tax_table(taxdat)
```

```
rownames(otudat) <- taxdat[rownames(taxdat) %in% rownames(otudat), taxa_level]
rownames(taxdat) <- taxdat[, taxa_level]
taxdat <- tax_table(taxdat)
taxa_names(physeq) <- taxa_names(taxdat)
tax_table(physeq) <- taxdat
otu_table(physeq) <- otudat

}


#============================================#
} else if (taxa_level == "Genus") {

    physeq = tax_glom(physeq = ps, taxrank = taxa_level, NArm = NArm)
    taxdat = tax_table(physeq)[, seq_along(rank.names[1:which(rank.names ==
taxa_level)])]

   taxdat = taxdat[complete.cases(taxdat),] %>% as.data.frame
    otudat = otu_table(physeq)

# take care of the uncultured genus
taxdat[,6] = ifelse(taxdat[,6] %in% c("uncategorized", NA, "uncultured", "unassigend",
"", " "),
       paste0("[", taxdat[,length(rank.names[1:which(rank.names==taxa_level)])-1], "]",
       "_", taxdat[,taxa_level]), taxdat[,taxa_level])

gen1 = taxdat[, taxa_level] %>% as.vector
gen2  = taxdat[, taxa_level] %>% as.vector

    uni  = matrix(NA, ncol = length(gen2), nrow = length(gen1))
    for(i in seq_along(gen1)){
        for(j in seq_along(gen2)){
    uni[i, j] = ifelse(gen1[i] == gen2[j] , "TRUE", "FALSE")
    }
        }

rownames(uni) <-gen1
colnames(uni) <- gen2
uni[upper.tri(uni, diag = TRUE)] = 0 #get rid of diagonals and upper triangle

duplis = uni %>% reshape2::melt() %>% filter(value == "TRUE")

        if(dim(duplis)[[1]] > 0){#if there is not duplications, we can simply use the
        taxa names as the row name

        duplis = uni %>% reshape2::melt() %>% filter(value == "TRUE") %>%
dplyr::select(1)%>% unique() %>% unlist %>% as.vector
        taxdat = taxdat %>% mutate( uni= ifelse(taxdat[, taxa_level] %in% duplis,
                    paste0("[",
                    taxdat[,length(rank.names[1:which(rank.names==taxa_level)])-1], "]",
                    "_", taxdat[,taxa_level]), taxdat[,taxa_level]))

        taxdat[, taxa_level] = taxdat[, "uni"]
```

```
        taxdat[, "uni"] <- NULL

        taxdat <- as(taxdat, "matrix")

        rownames(otudat) <- taxdat[rownames(taxdat) %in% rownames(otudat), taxa_level]
        rownames(taxdat) <- taxdat[taxdat[,taxa_level] %in% rownames(otudat), taxa_level]
        taxdat <- as.matrix(taxdat)
        taxdat <- tax_table(taxdat)
        taxa_names(physeq) <- taxa_names(taxdat)
        tax_table(physeq) <- taxdat
        otu_table(physeq) <- otudat

        } else {

        taxdat <- as.matrix(taxdat)
        taxdat <- tax_table(taxdat)
        rownames(otudat) <- taxdat[rownames(taxdat) %in% rownames(otudat), taxa_level]
        rownames(taxdat) <- taxdat[, taxa_level]
        taxdat <- tax_table(taxdat)
        taxa_names(physeq) <- taxa_names(taxdat)
        tax_table(physeq) <- taxdat
        otu_table(physeq) <- otudat
       }

} else {


physeq = tax_glom(physeq = ps, taxrank = taxa_level, NArm = TRUE)
    taxdat = tax_table(physeq)[, seq_along(rank.names[1:which(rank.names ==
taxa_level)])]

taxdat = taxdat[complete.cases(taxdat),] %>% as.data.frame
otudat = otu_table(physeq)

spec1 = taxdat[, taxa_level] %>% as.vector
spec2  = taxdat[, taxa_level] %>% as.vector

    uni  = matrix(NA, ncol = length(spec2), nrow = length(spec1))
    for(i in seq_along(spec1)){
        for(j in seq_along(spec2)){
    uni[i, j] = ifelse(spec1[i] == spec2[j] , "TRUE", "FALSE")
    }
        }

rownames(uni) <-spec1
colnames(uni) <- spec2
uni[upper.tri(uni, diag = TRUE)] = 0 #get rid of diagonals and upper triangle

duplis = uni %>% reshape2::melt() %>% filter(value == "TRUE")

if(dim(duplis)[[1]] > 0){#if there is not duplications, we can simply use the taxa names
as the row name
```

```
    duplis = uni %>% reshape2::melt() %>% filter(value == "TRUE") %>% dplyr::select(1)%>%
unique() %>% unlist %>% as.vector
taxdat = taxdat %>% mutate( uni= ifelse(taxdat[, taxa_level] %in% duplis,
                    paste(taxdat[,length(rank.names[1:which(rank.names==taxa_level)])-1],
                    "_", taxdat[,taxa_level]), taxdat[,taxa_level]))

taxdat[, taxa_level] = taxdat[, "uni"]
taxdat[, "uni"] <- NULL
taxdat <- as.matrix(taxdat)
rownames(otudat) <- taxdat[rownames(taxdat) %in% rownames(otudat), taxa_level]
rownames(taxdat) <- taxdat[, taxa_level]
taxdat <- tax_table(taxdat)
taxa_names(physeq) <- taxa_names(taxdat)
tax_table(physeq) <- taxdat
otu_table(physeq) <- otudat
} else {

taxdat <- as.matrix(taxdat)
taxdat <- tax_table(taxdat)
rownames(otudat) <- taxdat[rownames(taxdat) %in% rownames(otudat), taxa_level]
rownames(taxdat) <- taxdat[, taxa_level]
taxdat <- tax_table(taxdat)
taxa_names(physeq) <- taxa_names(taxdat)
tax_table(physeq) <- taxdat
otu_table(physeq) <- otudat
}
#ps = phyloseq(otu_table(otudat, taxa_are_rows = T), tax_table(as.matrix(taxdat)),
sample_data(physeq))


}
return(physeq)
    }
```

5.2. Graph-based analysis for beta diversity

```
#For total none-rarefied dataset
pst.spec <- gloomer(ps = pst, taxa_level = "Species", NArm = TRUE)
ps_total = prune_taxa(taxa_sums(pst.spec)>1000, pst.spec)#filtering the taxa based on
total sum
sample_data(ps_total)$sampleID <- rownames(sample_data(ps_total))
net <- make_network(ps_total, max.dist = 0.35, distance = "bray", type = "samples")
sampledata <- sample_data(ps_total) %>% data.frame
sampledata$sampleID <- rownames(sampledata)
V(net)$id <- sampledata[names(V(net)), "status"] %>% as.vector
V(net)$sample <-  rownames(sampledata)[rownames(sampledata) %in% names(V(net))] %>%
as.vector
V(net)$sample_type <- sampledata[names(V(net)), "sample_type"] %>% as.vector

#graph permutational test
graph.test <- graph_perm_test(ps_total, sampletype = "sample_type", grouping =
"sampleID", distance = "bray", type = "mst",
```

```
                              nperm = 1000)
graph.test$pval

V(graph.test$net)$status <- sampledata[names(V(graph.test$net)), "status"] %>% as.vector
plotNet1=plot_test_network(graph.test) + theme(legend.text = element_text(size = 8),
        legend.title = element_text(size = 9)) + geom_nodes(size = 4, aes(color =
        sampletype, shape = status))+
scale_color_manual(values = c("darkorchid3", "springgreen2"))

plotPerm1=plot_permutations(graph.test) + geom_text(aes(label = "P < 0.01", x = 76, y
=20), color = "red")

net.grid = grid.arrange(ncol = 2,  plotNet1, plotPerm1) + geom_col(inherit.aes = F, color
= "red")

ggsave("./graph_perm_total.jpeg", plot = net.grid, device = "jpeg", width = 15, beight =
10, dpi = 300)
```

5.3. Statisticl analysis for Beta diversity index: a distance-based redundancy analysis (dbRDA)

```
#Now we break the dataset into digesta and mucuse and do the same analysis
ps.dig = subset_samples(ps_rar, sample_data(ps_rar)$sample_type == "Digesta")
ps.muc = subset_samples(ps_rar, sample_data(ps_rar)$sample_type == "Mucose")

ps.dig.log = transform_sample_counts(ps.dig, function(x) log(1+x))
ps.muc.log = transform_sample_counts(ps.muc, function(x) log(1+x))


#Calculating Bray-Curtis dissimilarity coefficeints
bray.dist.log = phyloseq::distance(ps.log, method = "bray")#distance on dataset with both
digesta and mucosal samples

#digesta
bray.dist.dig.log = phyloseq::distance(ps.dig.log, method = "bray")

#mucusa
bray.dist.muc.log = phyloseq::distance(ps.muc.log, method = "bray")
```

5.3.1. Checking the dispersion of variance around the centroid (variance homeoginiety test)

```
#test for the disperssion of the variance around the centroids

#set the age of the animal as the random variable
set.seed(1990)
h <- with(data = data.frame(sample_data(ps.log)), how(blocks = age, nperm = 9999))

##total data
```

```
#Now we do a Homogeneity of dispersion test
set.seed(10)
bray.disp <- vegan::betadisper(bray.dist.log, group = sample_data(ps.log)$sample_type,
                                 type = "centroid")#if the p-value is significant, it
                                 means that there is a significant difference in variance
                                 for any of the tested levels.
perm.test = permutest(bray.disp, permutation =h, pairwise = T)

p.val.perm = perm.test$tab$`Pr(>F)`[[1]]

disp.centroid = bray.disp$centroids %>% as.data.frame
disp.vectors = bray.disp$vectors %>% as.data.frame
eig.vals = bray.disp$eig

#to remove all open graphic devices if jpeg function doesn't save the picture
for(i in dev.list()[1]:dev.list()[length(dev.list())]){
  dev.off()
  }

jpeg( "./Beta/dispersion of variance_bray_total_sample.type.jpeg",  quality = 100)

plot(bray.disp, col = c("deepskyblue", "chocolate1", "darkgreen"),
    bty = "n",  las = 1,
     main = "Dispersion of variance around the centroids, \n bray, total dataset",
     sub=NULL,  xlab = sprintf("PCo1 [%s%%]", round(eig.vals/sum(eig.vals)*100,1)[1]),
      ylab = sprintf("PCo2 [%s%%]",
    round(eig.vals/sum(eig.vals)*100,1)[2])); text("P < 0.01",
        x = -0.14, y = -0.35, cex = 1.5, col = "red")

##digesta data
#set the age of the animal as the random variable
set.seed(1990)
h <- with(data = data.frame(sample_data(ps.dig.log)), how(blocks = age, nperm = 9999))
#How we do a Homogeneity of dispersion test
set.seed(10)
bray.disp.dig <- vegan::betadisper(bray.dist.dig.log, group =
sample_data(ps.dig.log)$status,
                                 type = "centroid")#if the p-value is significant, it
                                 means that there is a significant difference in variance
                                 for any of the tested levels.
perm.test = permutest(bray.disp.dig, permutation =h, pairwise = T)


#since the betadisp is not significant, it is obvous that the variance around the
centroids are rather homogenusly dispersed for all groups
# and therefore the difference of our dbrda comes mainly from the treatments
disp.centroid = bray.disp.dig$centroids %>% as.data.frame
disp.vectors = bray.disp.dig$vectors %>% as.data.frame
eig.vals = bray.disp.dig$eig
p.val = perm.test$tab$`Pr(>F)`[1]

#tor remove all open graphic devices
```

```
for(i in dev.list()[1]:dev.list()[length(dev.list())]){
      dev.off()
}

jpeg( "./Beta/dispersion of variance_bray_digesta.jpeg", quality = 100)

plot(bray.disp.dig, col = c("deepskyblue", "chocolate1", "darkgreen"), bty = "n",
    las = 1, main = "Dispersion of variance around the centroids of Bray \n distance
    matrix for different diarrhea status in digesta", sub=NULL,
     xlab = sprintf("PCoA1 [%s%%]", round(eig.vals/sum(eig.vals)*100,0)[1]),
    ylab = sprintf("PCoA2 [%s%%]", round(eig.vals/sum(eig.vals)*100,1)[2])); text(glue("P
    = {round(p.val, 2)}"),
                                                                     x = 0.2, y = -0.32,
                                                                     cex = 1.5, col =
                                                                     "red")
rm(disp.centroid, disp.vectors, eig.vals, p.val, bray.disp.dig, perm.test, h)

##mucosal data
#set the age of the animal as the random variable
set.seed(1990)
h <- with(data = data.frame(sample_data(ps.muc.log)), how(blocks = age, nperm = 9999))
#How we do a Homogeneity of dispersion test
set.seed(10)
bray.disp.muc <- vegan::betadisper(bray.dist.muc.log, group =
sample_data(ps.muc.log)$status,
                                 type = "centroid")#if the p-value is significant, it
                                 means that there is a significant difference in variance
                                 for any of the tested levels.
perm.test = permutest(bray.disp.muc, permutation =h, pairwise = T)

#since the betadisp is not significant, it is obvous that the variance around the
centroids are rather homogenusly dispersed for all groups
# and therefore the difference of our dbrda comes mainly from the treatments
disp.centroid = bray.disp.muc$centroids %>% as.data.frame
disp.vectors = bray.disp.muc$vectors %>% as.data.frame
eig.vals = bray.disp.muc$eig
p.val = perm.test$tab$`Pr(>F)`[1]

#to remove all open graphic devices
for(i in dev.list()[1]:dev.list()[length(dev.list())]){
  dev.off()
}

jpeg( "./Beta/dispersion of variance_bray_muc.jpeg", quality = 100)

plot(bray.disp.muc, col = c("deepskyblue", "chocolate1", "darkgreen"), bty = "n",
    las = 1, main = "Dispersion of variance around the centroids of Bray\n distance
    matrix for different diarrhea status in mucose", sub=NULL,
     xlab = sprintf("PCoA1 [%s%%]", round(eig.vals/sum(eig.vals)*100,0)[1]),
    ylab = sprintf("PCoA2 [%s%%]", round(eig.vals/sum(eig.vals)*100,1)[2])); text(glue("P
    = {round(p.val, 2)}"),
                                                                     x = -0.25, y = -0.25,
                                                                     cex = 1.5, col =
                                                                     "red")
```

```
rm(disp.centroid, disp.vectors, eig.vals, p.val, bray.disp.muc, perm.test, h)
```

5.3.2. dbRDA model

```
#Whole dataset
set.seed(1990)
h <- with(data = data.frame(sample_data(ps.log)), how(blocks = age, nperm = 9999))

bray.dbrda = dbrda(t(otu_table(ps.log)) ~ status + Condition(round, age),
                        dist = "bray", permutations=h, data =
                        sample_data(ps.log)%>%data.frame)
#Sex not sig, so reduced the model

bray.dbrda

permutest(x = bray.dbrda, by = "terms", permutations = h)

#Digesta
set.seed(1990)
h <- with(data = data.frame(sample_data(ps.dig.log)), how(blocks = age, nperm = 9999))

bray.dbrda.dig = dbrda(t(otu_table(ps.dig.log)) ~ status +  Condition(round, age) ,
                        distance = "bray", permutations=h, data =
                        sample_data(ps.dig.log)%>%data.frame)#sex was not significant so
                        it has eventually been removed

bray.dbrda.dig


permutest(x = bray.dbrda.dig, by = "terms", permutations = h)

#Mucosal dataset
set.seed(1990)
h <- with(data = data.frame(sample_data(ps.muc.log)), how(blocks = age, nperm = 9999))

bray.dbrda.muc = dbrda(t(otu_table(ps.muc.log)) ~  status + Condition(round, age),
                        distance = "bray", permutations=h, data =
                        sample_data(ps.muc.log)%>%data.frame)#sex was not significant so
                        it has eventually been removed


bray.dbrda.muc


permutest(x = bray.dbrda.muc, by = "terms", permutations = h)
```

5.3.3. Plotting the model extracts

```r
# Make the plot out of the model, which is the variation explained only by the terms

#digesta
score.site = vegan::scores(bray.dbrda.dig, display = "sites") %>% as.data.frame
score.centroid = vegan::scores(bray.dbrda.dig, display = "cn")%>% as.data.frame
rownames(score.centroid)<- levels(sample_data(ps.dig.log)$status)
score.centroid

eig.vals = bray.dbrda.dig$CCA$eig
inertia.total = bray.dbrda.dig$tot.chi #total variation (inertia) explained.
#this number should be used as the denominator for measuring the amount of variance out
of totoal variance wxplained by each dbrda

#Digesta
score.site %>% ggplot(aes(dbRDA1, dbRDA2, color = sample_data(ps.dig.log)$status)) +
geom_point(size =6 ) +
geom_hline(yintercept = 0, lty = 2, alpha =0.5) +
geom_vline(xintercept = 0, lty = 2, alpha = 0.5)+
coord_fixed() +
scale_color_manual(values = c(alpha(colour = "deepskyblue", 0.8),
                              alpha(colour = "chocolate1", 0.8), alpha(colour =
                              "darkgreen", 0.8) )) +
theme_bw() +
scale_y_continuous(na.value = c(-2, 3), n.breaks = 10) +
scale_x_continuous(na.value = c(-1, 1), n.breaks = 10) +
labs(col="Diarrheal status") +
xlab(label = paste("dbRDA1 [", round(eig.vals[[1]]/sum(eig.vals)*100, 1),
                    "% of fitted and",
                   round(eig.vals[[1]]/inertia.total*100, 1),
                   "% of total variation]")) +
ylab(label = paste("dbRDA2 [", round(eig.vals[[2]]/sum(eig.vals)*100, 1),
                   "% of fitted and", round(eig.vals[[2]]/inertia.total*100, 1),
                   "of total variation]")) +
theme(axis.title = element_text(size = 10),
      text = element_text(size = 13, face = "bold"),
      axis.text.x =element_text(size =10, face = "bold"),
axis.text.y =element_text(size =10, face = "bold")) +
ggtitle(label = "dbRDA plot of Bray in digesta")  +
stat_ellipse(aes(group = sample_data(ps.dig.log)$status), fill = "yellow", show.legend =
F,
                            type = "t", level = 0.9,
                            lty = 2, geom = "polygon", alpha = 0.07)

ggsave("./Beta/bray.dbRDA.dig.jpeg", height = 8, width = 9, dpi =300)

#Mucus

score.site = vegan::scores(bray.dbrda.muc, display = "sites") %>% as.data.frame
score.centroid = vegan::scores(bray.dbrda.muc, display = "cn")%>% as.data.frame
rownames(score.centroid) <- levels(sample_data(ps.muc.log)$status)
score.centroid

eig.vals = bray.dbrda.muc$CCA$eig
```

```
inertia.total = bray.dbrda.muc$tot.chi


score.site %>% ggplot(aes(dbRDA1, dbRDA2, color = sample_data(ps.muc.log)$status)) +
geom_point(size =6 ) +
geom_hline(yintercept = 0, lty = 2, alpha =0.5) +
geom_vline(xintercept = 0, lty = 2, alpha = 0.5)+
coord_fixed() +
scale_color_manual(values = c(alpha(colour = "deepskyblue", 0.8),
                              alpha(colour = "chocolate1", 0.8),
                              alpha(colour = "darkgreen", 0.8) )) +
theme_bw() +
scale_y_continuous(na.value = c(-2, 3), n.breaks = 10) +
scale_x_continuous(na.value = c(-1, 1), n.breaks = 10) +
labs(col="Diarrheal status") +
xlab(label = paste("dbRDA1 [", round(eig.vals[[1]]/sum(eig.vals)*100, 1),
                    "% of fitted and",
                   round(eig.vals[[1]]/inertia.total*100, 1),
                   "% of total variation]")) +
ylab(label = paste("dbRDA2 [", round(eig.vals[[2]]/sum(eig.vals)*100, 1),
                   "% of fitted and", round(eig.vals[[2]]/inertia.total*100, 1),
                   "of total variation]")) +
theme(axis.title = element_text(size = 10),
      text = element_text(size = 13, face = "bold"),
      axis.text.x =element_text(size =10, face = "bold"),
axis.text.y =element_text(size =10, face = "bold")) +
ggtitle(label = "dbRDA plot of Bray in mucose")  +
stat_ellipse(aes(group = sample_data(ps.muc.log)$status), fill = "yellow", show.legend =
F,
                             type = "t", level = 0.9,
                             lty = 2, geom = "polygon", alpha = 0.07)

ggsave("./Beta/bray.dbRDA.muc.jpeg", height = 8, width = 9, dpi =300)
```

6. Differential abunance analysis: DESeq2

6.1. Stacked barplot

```
#Aglomerating the taxa

phylu.dig =  gloomer(ps.dig, taxa_level =  "Phylum", NArm = TRUE)
phylu.muc =  gloomer(ps.muc, taxa_level =  "Phylum", NArm = TRUE)

#Barplot of relative abundance of phylum in digesta
trans.dig.phyl <- merge_samples(phylu.dig, "status")
relabund.dig.phyl <- transform_sample_counts(trans.dig.phyl, function(x) x / sum(x)*100)

#choosing colors
phylcol=c( "deepskyblue",'springgreen3','snow3','burlywood4', 'cadetblue', 'darkblue',
          'cornflowerblue','deeppink2','orangered',  'dimgrey', 'red','limegreen',
```

```
       'cyan1','darkmagenta', 'purple', 'cyan4', 'gold')

#checking which seed number samples the best color for phylum
r = length(tax_table(phylu.dig)[,2] %>% unique)
c = 30
phylcol2 = matrix(NA, ncol = c, nrow = r)
plot.list <- vector(mode = "list", length = length(1:c))

for(i in 1:c) {
set.seed(i)

phylcol2[,i] =  sample(phylcol, size = length(tax_table(phylu.dig)[,2] %>% unique),
replace = F) %>% factor %>% levels

 plots <- plot_bar(relabund.dig.phyl, fill="Phylum")+
    scale_fill_manual(values = phylcol2[,i]) + xlab("Diarrheal Status") +
    ggtitle(label = paste("Seed", sep = "=", i))  +
            theme_bw() +
            theme( legend.position = "right", text = element_text(size =15, face =
            "bold")) + coord_flip()
    plot.list[[i]] <- plots
}

set.seed(41)#number 24 was the best
phylcol2 = sample(phylcol, size = length(tax_table(phylu.dig)[,2] %>% unique), replace =
F) %>% factor %>% levels


plot_bar(relabund.dig.phyl, fill="Phylum")+
            scale_fill_manual(values = phylcol2) + xlab("Diarrheal Status") +
            ylab("Relative abundance of taxa > 0.01% of total abundance in digesta, %") +
            theme_bw() + theme(legend.box.spacing =  unit(x = 0.5, units = "cm"),
            legend.spacing.y = unit(x = 0.5, "cm") ) +
            theme( legend.position = "right", text = element_text(size =15, face =
            "bold")) + coord_flip()

ggsave("./Beta/barplot_phylum_dig.newCol.jpeg", dpi = 300, width = 10, height = 4.5)

#Barplot of relative abundance of phylum in mucos
trans.muc.phyl <- merge_samples(phylu.muc, "status")
relabund.muc.phyl <- transform_sample_counts(trans.muc.phyl, function(x) x / sum(x)*100)

plot_bar(relabund.muc.phyl, fill="Phylum")  +
            scale_fill_manual(values = phylcol2) + xlab("Diarrheal Status") +
            ylab("Relative abundance of taxa > 0.01% of total abundance in mucus, %") +
            theme_bw() +
            theme( legend.position = "right", text = element_text(size =15, face =
            "bold")) + coord_flip()

ggsave("./Beta/barplot_phylum_muc.newCol.jpeg", dpi = 300, width = 10, height = 4.5)
```

6.2. DESeq2

```
#glooming the taxa for digesta dataset without rarefaction
pst.dig = subset_samples(physeq = pst, sample_type == "Digesta")#we use unrarefied object
for DESeq

phyl.dig =  gloomer(pst.dig, taxa_level =  "Phylum", NArm = TRUE)
genus.dig = gloomer(pst.dig, taxa_level = "Genus", NArm = TRUE)
spec.dig = gloomer(pst.dig, taxa_level = "Species", NArm = TRUE)


#glooming the taxa for mucusal dataset
pst.muc = subset_samples(physeq = pst, sample_type == "Mucose")#we use unrarefied object
for DESeq

phyl.muc =  gloomer(pst.muc, taxa_level = "Phylum", NArm = TRUE)
genus.muc = gloomer(pst.muc, taxa_level = "Genus", NArm = TRUE)

##Digesta DESeq

#converting phylosq to deseq
phyl.dds.dig<-phyloseq_to_deseq2(phyl.dig, design = ~  status)
gen.dds.dig <- phyloseq_to_deseq2(genus.dig, design = ~ status)

#calculate geometric means prior to estimate size factors
gm.mean = function(x, na.rm= TRUE) {
    exp(sum(log(x[x>0]), na.rm=na.rm)/length(x))
}

##Phylum level
geo.mean = apply(counts(phyl.dds.dig), 1, gm.mean)
phyl.dds.dig = estimateSizeFactors(phyl.dds.dig, geoMeans = geo.mean)
phyl.dds.dig<-DESeq(phyl.dds.dig, test = "Wald", fitType = "parametric")

#Genus level
geo.mean = apply(counts(gen.dds.dig), 1, gm.mean)
gen.dds.dig = estimateSizeFactors(gen.dds.dig, geoMeans = geo.mean)
gen.dds.dig<-DESeq(gen.dds.dig, test = "Wald", fitType = "parametric")



#mucose
#converting phylosq to deseq
phyl.dds.muc<-phyloseq_to_deseq2(phyl.muc, design = ~  status)
gen.dds.muc <- phyloseq_to_deseq2(genus.muc, design = ~ status)

#calculate geometric means prior to estimate size factors
gm.mean = function(x, na.rm= TRUE) {
    exp(sum(log(x[x>0]), na.rm=na.rm)/length(x))
}

geo.mean = apply(counts(phyl.dds.muc), 1, gm.mean)
phyl.dds.muc = estimateSizeFactors(phyl.dds.muc, geoMeans = geo.mean)
```

```r
phyl.dds.muc<-DESeq(phyl.dds.muc, test = "Wald", fitType = "parametric")

geo.mean = apply(counts(gen.dds.muc), 1, gm.mean)
gen.dds.muc = estimateSizeFactors(gen.dds.muc, geoMeans = geo.mean)
gen.dds.muc<-DESeq(gen.dds.muc, test = "Wald", fitType = "parametric")
```

6.3. visualizing DESeq results: waterfall and volcano plots

```r
library(ggrepel)
#Waterfall plot for phylum

sigtabphyl = phyl.muc.DiarNoInfl.vs.NoDiar %>% rownames_to_column("Phylum")

#plotting for the phylum alone
theme_set(theme_bw())
phylcol=c( "deepskyblue",'springgreen3','snow3','burlywood4', 'cadetblue', 'darkblue',
          'cornflowerblue','deeppink2','orangered', 'dimgrey', 'red','limegreen',
        'cyan1','darkmagenta', 'purple', 'cyan4', 'gold')

colindex = data.frame(color = phylcol[1:length(unique(tax_table(phyl.dig)[,2]))],
                      phylum = sort(unique(tax_table(phyl.dig)[,2])))

phyla = unique(data.frame(tax_table(phyl.dig)[,2])) %>% pull
colors = c()
for(i in phyla){
   colors[i] = colindex[colindex$Phylum == i,1]
}


#filtering results above 0.01 padjust
alpha = 0.05
sigtabphyl = sigtabphyl[sigtabphyl$padj <=alpha,]
# Phylum order
x = tapply(sigtabphyl$log2FoldChange, sigtabphyl$Phylum, function(x) max(x))
x = sort(x, TRUE)
sigtabphyl$Phylum = factor(as.character(sigtabphyl$Phylum), levels=names(x))

ggplot(sigtabphyl, aes(y=Phylum, x=log2FoldChange), stroke = 0.5) +
  geom_vline(xintercept = 0.0,
             color = "orange", size = 0.5, lty = 2) +
  geom_point(aes(fill = Phylum), alpha = 0.6, size = 14, color = "black", shape = 21,
  stroke = 0.5) +
  theme(legend.key.width = unit(0.1, "cm"),
      legend.key.height = unit(0.1, "cm"),
      legend.key.size = unit(x = 0, units = "cm" ), legend.box = unit(20, "cm"),
        title = element_text(size = 15, color = "black", face = "bold"),
        text = element_text(size =15, face = "bold"),
        axis.text.x = element_text(angle = -90, hjust = 0,
        vjust=0.5, size = 12, face = "bold"),
        axis.text.y = element_text(size = 13, face = "italic" )) +
           scale_x_continuous(limits = c(-8, 3.5), n.breaks = 10) +
```

```
           ggtitle("Log2FoldChange of Phylum in mucus,
DiarNoInfl vs. NoDiar, no filt for logFC") +
scale_fill_manual(values = colors[names(colors) %in% sigtabphyl$Phylum])

ggsave("./deseq2/mucus/difabund_muc_DiarNoInfl_vs_NoDiar.jpeg", device = "jpeg", dpi =
300)

rm(sigtabphyl, alpha, x, colors, colindex, phyla)


# Figures for genus and Phylum in mucose. The same was doen for digesta results

theme_set(theme_bw())

spec.taxa = tax_table(genus.muc) %>% as.matrix

sigtabspec = cbind(as(gen.muc.DiarInfl.vs.DiarNoInfl, "data.frame"),
                   as(spec.taxa[rownames(spec.taxa) %in%
                   rownames(gen.muc.DiarInfl.vs.DiarNoInfl),], "matrix"))

alpha = 0.05


#filtering out the taxa below 2 LFC
sigtabspec = sigtabspec[abs(sigtabspec$log2FoldChange)>2,]

#a costumized color scheme
phylcol= c( "deepskyblue",'springgreen3','snow3','burlywood4', 'cadetblue', 'darkblue',
          'cornflowerblue','deeppink2','orangered', 'dimgrey', 'red','limegreen',
        'cyan1','darkmagenta', 'purple', 'cyan4', 'gold')

colindex = data.frame(color = phylcol[1:length(unique(tax_table(genus.muc)[,2]))], phylum
= sort(unique(tax_table(genus.muc)[,2])))


phyla = unique(data.frame(tax_table(genus.muc)[,2])) %>% pull
colors = c()
for(i in phyla){
   colors[i] = colindex[colindex$Phylum == i,1]
}


#filtering results above 0.01 padjust

sigtabspec = sigtabspec[sigtabspec$padj <=alpha,]

# Phylum order
x = tapply(sigtabspec$log2FoldChange, sigtabspec$Phylum, function(x) max(x))
x = sort(x, TRUE)
sigtabspec$Phylum = factor(as.character(sigtabspec$Phylum), levels=names(x))


#Species reorder
```

```
x = tapply(sigtabspec$log2FoldChange, sigtabspec$Genus, function(x) max(x))
x = sort(x, TRUE)
sigtabspec$unique = factor(as.character(sigtabspec$Genus), levels=names(x))

ggplot(sigtabspec, aes(y=unique, x=log2FoldChange), stroke = 0.5) +
  geom_vline(xintercept = 0.0, color = "orange", size = 0.5, lty = 2) +
  geom_point(aes(fill = Phylum), alpha = 0.6, size = 10, color = "black", shape = 21,
  stroke = 0.5)+
  theme(legend.text = element_text(face = "bold"),
        axis.text.x = element_text(angle = -90, hjust = 0, vjust=0.5, size = 12, face =
        "bold"),
        axis.text.y = element_text(size = 14, face = "italic"), title = element_text(face
        = "bold") ) +
        ggtitle("Log2FoldChange of Genus in DiarInfl vs. DiarNoInfl, \n muc logFC
        filtered") +
        scale_x_continuous(limits = c(-7, 8),n.breaks = 10) +
        scale_y_discrete(expand = c(0.0005,0.9))+
geom_text(aes(label = round(log2FoldChange,2)), size = 3 )+
        scale_fill_manual(values = colors[names(colors)%in% sigtabspec$Phylum]) +
        ylab("Species")
           #+ geom_text(aes( label = -log10(padj) %>% round(1)),inherit.aes = TRUE,
           nudge_y = 0.4, color = "black")
ggsave("./deseq2/mucus/difabund_gen_DiarInfl_vs_DiarNoInfl_muc.jpeg", device = "jpeg",
dpi  = 300, height = 16, width = 10)
```

```
#Volcano plot

alpha = 0.05
gen.dat = results(gen.dds.muc, contrast =  c("status", "DiarNoInfl",
"NoDiar"))%>%data.frame
gen.dat = gen.dat[complete.cases(gen.dat),]
gen.dat$Significant = ifelse(gen.dat$padj < alpha, paste0("FDR < ", alpha), "Not Sig")
%>%
factor(levels = c("FDR < 0.05", "Not Sig"))

gen.taxa = tax_table(genus.muc) %>% as.matrix

sigtabgen = cbind(as(gen.dat, "data.frame"),
                   as(gen.taxa[rownames(gen.taxa) %in% rownames(gen.dat),], "matrix"))

#a costumized color scheme
phylcol=c('coral4', "deeppink",'brown2','antiquewhite4', 'cornflowerblue', 'plum4',
          'darkgoldenrod3','aquamarine4', 'yellow', 'red', 'darkblue', 'Maroon', 'Gray',
        'steelblue2','darkgreen', 'tomato1', 'cyan4', 'magenta')

colindex = data.frame(color = phylcol[1:length(unique(tax_table(genus.muc)[,2]))], phylum
= sort(unique(tax_table(genus.muc)[,2])))


phyla = unique(data.frame(tax_table(genus.muc)[,2])) %>% pull
colors = c()
for(i in phyla){
   colors[i] = colindex[colindex$Phylum == i,1]
```

```
}



#filtering out the taxa below 2 LFC
#sigtabspec = sigtabspec[abs(sigtabspec$log2FoldChange)>2,]

sigtabgen %>% group_by(log2FoldChange) %>% arrange(desc(log2FoldChange)) %>%
ggplot(aes(x = log2FoldChange, y = -log10(pvalue), label = Genus)) +
geom_hline(yintercept = -log10(sigtabgen[sigtabgen$Significant == "Not Sig","pvalue"])
%>%
           max, color = alpha("red",0.5), lty = 2) +
geom_vline(xintercept = 0, color = alpha("black", 0.3))  +
geom_point(data = sigtabgen[sigtabgen$Significant == "Not Sig",],
           aes(x = log2FoldChange, y = -log10(pvalue)),
           color = alpha("darkgreen", 0.6),  size = 2)  +
theme_bw(base_size = 12) +
theme(legend.position= "right",
      text = element_text(size = 15, face = "bold")) +
geom_point(data = sigtabgen[sigtabgen$Significant == "FDR < 0.05",],
    aes(x = log2FoldChange, y = -log10(pvalue),
        fill = Phylum), size = 6, alpha = 0.5,
        color = "black", shape = 21, stroke = 0.5) +
scale_fill_manual(values = colors[names(colors) %in%
                sigtabspec[sigtabgen$Significant == "FDR < 0.05", "Phylum"]]) +
geom_text_repel( nudge_y = 0.15, nudge_x = -.5,
               data= top_n(sigtabgen[sigtabgen$Significant == "FDR < 0.05" &
               sigtabgen$log2FoldChange < -2,],
               n =  -10, wt = pvalue),
               aes(label = Genus),
               size = 2.5,
               box.padding = unit( 0.4, units ="lines"),
               point.padding = unit(0.4, "lines"), max.overlaps = 20)  +
geom_text_repel(nudge_y = 0, nudge_x =0.5,
               data= top_n(sigtabgen[sigtabgen$Significant == "FDR < 0.05" &
               sigtabgen$log2FoldChange > 2,],
               -10, pvalue),
               aes(label = Genus), size = 2,
               box.padding = unit( 0.4, units ="lines"),
               point.padding = unit(0.4, "lines"), max.overlaps = 20)+
geom_text( aes(x = 4, y =0, label = "Not Sig"), color = "red", size = 2.5) +
geom_text(aes(x = 4, y =5, label = "FDR < 0.05"), color = "red", size = 2.5)+
ggtitle (label = "Volcano Plot of the top 10 most significant log2FoldChange\n
Genus for DiarNoInfl vs. NoDiar in Mucusa") + scale_y_continuous(limits = c(0, 17),
n.breaks = 5) +
scale_x_continuous(limits = c(-7.5, 6), n.breaks = 10) +
guides(size = "none")

ggsave("./deseq2/mucus/volc_gen_DiarNoInfl_vs_NoDiar_muc.jpeg", device = "jpeg", dpi =
300)
```

7. Linear regression for chemical data: Generalized Linear Mixed Effect Model

```
library(nlme)
library(lme4)
library(lmerTest)
library(car)
library(lsmeans)
library(postHoc)
library(multcomp)
library(glue)

#setting up a dataset for chemical data
chem.ccd = sample_data(ps_rar)[sample_data(ps_rar)$sample_type == "Digesta",c(1:37)]
chem.ccd <- chem.ccd[complete.cases(chem.ccd),] %>% data.frame

# A custume function for the model that extracts all model varaibles
glmer.helper <- function(df, response, formu, pair.form, plot.form, n.round, st.seed =
10){
  models <-list()
  summeries <- list()
  emms <- list()
  contrs <- list()
  plots <- list()
  pacman::p_load(nlme, lme4, lmerTest, car, lsmeans, postHoc, multcomp, glue)

#a function to round numbers and give you numeric values with your desired number of
digits. E.g. if you want your table to show numbers in 3 digits, you can uniformly round
your table to get numbers in 3 digits etc.
rounder <- function(x, n.round = 3) {
x = as.numeric(x)

if (grepl(x = round(x, n.round), pattern = ".", fixed = T) &
trunc(x) != 0  &
nchar(trunc(abs(x))) >= n.round) {

 x = round(x)

}  else if (trunc(x) == 0 &
nchar(round(abs(x), digits = n.round-1)) == n.round ){

 x = paste0(round(x, digits = n.round-1), ".0")

} else if (trunc(x) == 0 &
nchar(round(abs(x), digits = n.round-1)) == n.round +1){

 x = round(x, digits = n.round-1)

} else if (trunc(x) == 0 &
nchar(round(abs(x), digits = n.round-1)) < n.round ){

 x = paste0(round(x, digits = n.round-1), ".0")
```

```r
} else if (trunc(x) !=0 &
 nchar(round(abs(x), digits = n.round-1)) == n.round + 1){

  x = round(x, digits = n.round-1)

} else if (trunc(x) !=0 & nchar(round(abs(x))) == nchar(trunc(abs(x))) ){
 x = round(x, digits = n.round - nchar(trunc(abs(x))))

 if(nchar(abs(x))<n.round){

  x = paste0(x, ".0")

 } else {
  x = x
 }

}
return(x)
}

#here I vectorize my function to work for vectors as well
rounder <- Vectorize(FUN = rounder)

# model

  for(i in response){
  formula <- as.formula(paste(i, formu))
  formula.compare <- as.formula(pair.form)
  formula.plot <- as.formula(plot.form)

  ml <- glmer(formula = formula,
           data = df, family = Gamma(link = "log"),
           control = glmerControl(c("bobyqa", "bobyqa")))

#models
  models[[i]] <-ml
#summaries
  summeries[[i]] <- summary(ml)
#emmeans
  set.seed(seed = st.seed)

  emms[[i]] <- emmeans(ml, formula.compare,  type = "response")$emmeans %>%
  cld( adjust = "BH", Letters = letters) %>%
  data.frame() %>% group_by(segment)%>%
  mutate(check = ifelse( unique(.group) %>% length() > 1, "TRUE", "FALSE")) %>%
  mutate(resps = ifelse(check == "TRUE",
  glue("{rounder(response,3)} ({rounder(asymp.LCL,3)}-{rounder(asymp.UCL,3)}){.group}"),
  glue("{rounder(response,3)} ({rounder(asymp.LCL,3)}-{rounder(asymp.UCL,3)})"))) %>%
  dplyr::select( -df, -asymp.LCL, -asymp.UCL, -response, -SE, -.group, - check) %>%
  pivot_wider(names_from = status, values_from = resps)

#contrasts
```

```
  set.seed(seed = st.seed)

  contrs[[i]] <- emmeans(ml, formula.compare, type = "response")$contrasts %>%
  cld(Letters = letters, adjust = "BH") %>%
  data.frame() %>%
  mutate(contr = ifelse(contrast == "DiarNoInfl / DiarInfl",
  "DiarNoInfl vs. DiarInfl",
  ifelse(contrast== "NoDiar / DiarNoInfl",
  "NoDiar vs. DiarNoInfl",
  ifelse(contrast == "NoDiar / DiarInfl",
  "NoDiar vs. DiarInfl",
  ifelse(contrast == "DiarNoInfl / DiarInfl",
  "DiarNoInfl vs. DiarInfl",
  ifelse(contrast == "NoDiar / DiarInfl",
  "NoDiar vs. DiarInfl", "NoDiar vs. DiarNoInfl" )))))) %>%
  dplyr::select(-df, -null, -contrast,-p.value, -SE, -z.ratio, -ratio)%>%
  pivot_wider(names_from = contr, values_from = .group)

  #plots

  plots[[i]] <- emmip(ml, formula.plot) + theme_bw() + ggtitle(glue("Interaction plot for
{i}"))
  }

  structure(list(models, summeries, emms, contrs, plots))
}


df <- chem.ccd %>% filter(!pig_no %in% c("pig15", "pig7"))

xtabs(~   pig_no + segment, df)

#SCFA
resps <- colnames(chem.ccd)[c(9,12, 17:22)]

#Indoles and ammonia
resps <- colnames(chem.ccd)[c(23,24,25:28)]

#biogenic amines
resps <- colnames(chem.ccd)[c(29:31,33:37)]



test <- glmer.helper(df = df, response = resps, st.seed = 10,
pair.form = "pairwise ~ status | segment",
formu = "+0.000001 ~ status + segment + sex + status*segment + status * sex + segment *
sex +   (1|round)",
plot.form = "segment ~ status | sex")

samps <-list()

for(i in resps){
samps[[i]] = emmeans(test[[1]][[i]], ~ segment, type = "response", adjust = "bh") %>%
cld(Letters = letters,  confit.glht = "BH")%>%
```

```
data.frame() %>%
mutate(check = ifelse( unique(.group) %>% length() > 1, "TRUE", "FALSE")) %>%
  mutate(resps = ifelse(check == "TRUE",
  glue("{round(response,1)}({round(asymp.LCL)}-{round(asymp.UCL)}){.group}"),
  glue("{round(response,1)}({round(asymp.LCL)}-{round(asymp.UCL)})"))) %>%
  dplyr::select(-df, -response, -.group, -check, -asymp.UCL, -asymp.LCL,  -SE)
}

#writing the results
##SCFA
chems <- data.frame(SCFA = rep(resps, each = 2), rbind( test[[3]]$pH, test[[3]]$DM,
test[[3]]$SCFA,
test[[3]]$Acetate, test[[3]]$Propionate, test[[3]]$Butyrate, test[[3]]$Iso.acids,
test[[3]]$Valerate))



write.table(chems, './chemicals/scfa.tsv', sep = "\t")

#Indoles
chems <- data.frame(Indoles = rep(resps, each = 2), rbind(test[[3]]$Indoles,
test[[3]]$L.tryptophan,
test[[3]]$Indol.3.acetate,
test[[3]]$Indol.3.propionate,
test[[3]]$Indol.1.benzopyrrol,
test[[3]]$Indol.3.methylindole)) %>% dplyr::select(1,2,5,4,3)

write.table(chems, './chemicals/indoles.tsv', sep = "\t", row.names = F)

#biogenic amines
chems <- data.frame(Biogenic_amines = rep(resps, each = 2),
rbind(test[[3]]$Biogenic.amines,
test[[3]]$L.threonine,
test[[3]]$Agmatine,
test[[3]]$L.valine,
test[[3]]$L.lysine,
test[[3]]$Putrescine,
test[[3]]$Cadaverine,
test[[3]]$Ammonia)) %>% dplyr::select(1,2,5,4,3)

write.table(chems, './chemicals/biogenimc.amines.tsv', sep = "\t")
```

8. Heatmap associaiton between chemical and taxa data

```
chem.dat = chem.dat[complete.cases(chem.dat),] %>% rename(SCFA_total = "SCFA",
                                                          Indoles_total = "Indoles",
                                                          Biogenic_amines_total =
                                                          "Biogenic.amines")
```

```
#correlation heatmap between chemicals and the digesta genus
gen.dig <- gloomer(pst.dig, taxa_level = "Genus", NArm = TRUE)

pst.rel.gen = filter_taxa(gen.dig, function(x) sum(x>0)>0, TRUE) #filtering the zero
counts out
pst.rel.gen = transform_sample_counts(pst.rel.gen, function(x) x/sum(x))

chem.dat = chem.ccd[, colnames(chem.ccd) %in% c('SCFA','Acetate', 'Propionate',
'Butyrate', 'Iso.acids', 'Valerate', 'Indoles', 'L.tryptophan',
         'Indol.3.acetate', 'Indol.3.propionate', 'Indol.1.benzopyrrol',
         'Indol.3.methylindole', 'Phenol.4.methylphenol',
         'Biogenic.amines', 'L.threonine', 'Agmatine', 'DL.methionine', 'L.valine',
         'L.lysine', 'Putrescine', 'Cadaverine', 'Ammonia')]


chem.dat = chem.dat[complete.cases(chem.dat),] %>% rename(SCFA_total = "SCFA",
                                                         Indoles_total = "Indoles",
                                                         Biogenic_amines_total =
                                                         "Biogenic.amines")
chem.dat = chem.dat[, colSums(chem.dat)> 0]
log.chem.dat = apply(chem.dat, 2, function(x) log(x + 1))

asv.gen = as.matrix(otu_table(pst.rel.gen))

asv.gen = asv.gen[!rownames(asv.gen) %in% c("Unknown", "uncultured", "Uncultured",
"Unassigned", "NA"),]

asv.gen = t(asv.gen)
asv.gen = asv.gen[rownames(asv.gen) %in% rownames(log.chem.dat),]


#cor chem taxa

cor_main = Hmisc::rcorr(asv.gen, log.chem.dat, type = "spearman")
cor.chem = cor_main$r
cor.chem = cor.chem[rownames(cor.chem) %in% colnames(asv.gen), colnames(cor.chem) %in%
colnames(log.chem.dat)] #rows as taxa, cols as chemical data

#calculating the qvalues for the correlations
cor.pval = cor_main$P[rownames(cor_main$P) %in% rownames(cor.chem) , colnames(cor_main$P)
%in% colnames(cor.chem)]
cor.qval = p.adjust(cor.pval, method = "BH")
q.vals = matrix(cor.qval, ncol = ncol(cor.pval), nrow = nrow(cor.pval), dimnames =
list(rownames(cor.pval), colnames(cor.pval)))

#Adding significance signes to the qval matrix to be used in the heatmap later on
 q.vals[cor.qval <0.05] = "*"
q.vals[cor.qval >= 0.05] = ""

cor.scfa <-  cor.chem
```

```r
 cor.scfa.nodiar <-  cor.scfa[sample_data(gen.dig)$status == "NoDiar",]
 cor.scfa.diarinfl <-  cor.scfa[sample_data(gen.dig)$status == "DiarNoInfl",]
 cor.scfa.diarnoinfl <-  cor.scfa[sample_data(gen.dig)$status == "DiarInfl",]

cor.scfa.nodiar = cor.scfa.nodiar %>% data.frame() %>% mutate(status = as.factor(rep(x =
"NoDiar", dim(cor.scfa.nodiar)[[1]])))

cor.scfa.diarinfl = cor.scfa.diarinfl %>% data.frame() %>% mutate(status =
as.factor(rep(x = "DiarInfl", dim(cor.scfa.diarinfl)[[1]])))

cor.scfa.diarnoinfl = cor.scfa.diarnoinfl %>% data.frame() %>% mutate(status =
as.factor(rep(x = "DiarNoInfl", dim(cor.scfa.diarnoinfl)[[1]])))

cor_scfa = rbind(cor.scfa.nodiar, cor.scfa.diarinfl, cor.scfa.diarnoinfl)

cor_scfa$status <- factor(cor_scfa$status, levels =  c("NoDiar", "DiarNoInfl",
"DiarInfl"))
tax.df <- tax_table(gen.dig)[,2] %>% data.frame() %>% rownames_to_column("Species")
cor_scfa = cor_scfa %>% rownames_to_column("Species") %>% left_join(tax.df, by =
"Species")
cor_scfa = column_to_rownames(cor_scfa, var = "Species")


asvdat = as(cor_scfa[,1:21],"matrix")#in the aassay dataset, we add our correlation
matrix instead of the abundance matrix

taxadat = Biobase::AnnotatedDataFrame(cor_scfa)#taxa table
pdata = cor_scfa %>% data.frame

x = ExpressionSet(assayData = asvdat,  featureData = taxadat  )

#Adding phenotype data
pData(x) <- pdata

# Filtering based on row standard deviation and choosing the most variable 50 taxa
sds <- rowSds(Biobase::exprs(x)[,1:6])
o <- order(sds, decreasing = TRUE)[1:50]
h_1 <- hclust(dist(Biobase::exprs(x)[o,1:6]), method = "ward.D2")
h_2 <- hclust(dist(t(Biobase::exprs(x)[o,1:6])), method = "ward.D2")

#making a phylum annotation and it only accepts one column dataframe
row.annot = fData(x)[rownames(fData(x)) %in% rownames(Biobase::exprs(x)[o,]),] %>%
select(22,23)

#making color index for the phylum annotation

phylcol=c('coral4', 'cyan','gold', 'tomato1', 'cornflowerblue', 'plum4',
          'darkgoldenrod3','aquamarine4', 'cadetblue2', 'red', 'darkblue', 'Maroon',
          'Gray',
        'steelblue2','darkmagenta', 'antiquewhite4', "darkorange", 'darkgreen')
set.seed(26)
phylcol = sample(phylcol, size = length(unique(row.annot[,2])), replace = F)
phyl.col = data.frame(Phylum = unique(row.annot[,2]), phyl.col =
phylcol[1:length(unique(row.annot[,2]))])
```

```r
phyl.col = column_to_rownames(phyl.col, "Phylum") %>% as.matrix

status.col = data.frame(status = unique(row.annot[,1]), stat.col = c(alpha(colour =
"darkorange", 1),
                              alpha(colour = "deeppink", 1),
                              alpha(colour = "darkgreen", 0.7) ))
status.col = column_to_rownames(status.col, "status") %>% as.matrix




pheat.chem.scfa = pheatmap(Biobase::exprs(x)[o,1:6], annotation_row = pData(x)[o,] %>%
select(22,23),
                          cellheight = 12, annotation_colors = list(
                            status = status.col[,1],
                            Phylum = phyl.col[,1]),
                           cellwidth = 15, cutree_rows = 4, border_color = NA,
                           fontsize_number = 15, number_color = "black",
                           display_numbers = q.vals[rownames(q.vals) %in%
                           rownames(Biobase::exprs(x)[o,]), colnames(q.vals) %in%
                           colnames(exprs(x)[,1:6])], angle_col = 45,
                           Rowv = as.dendrogram(h_1), Colv = as.dendrogram(h_2),
                           cutcluster_rows = T, cluster_cols = F, col = brewer.pal(9,
                           "Greens"),
                           width = 10, height = 25, main = "spearman correlation of top
                           50 Genre \nand SCFA, clustered row ")
ggsave(plot = pheat.chem.scfa, "./heatmap/heatmap.SCFA.gen_50.jpeg", dpi = 750, height =
10, width = 8)
```

The end!

Email: farhad.m.panah@post.au.dk
