# Supplementary material for: Composition of mucus- and digesta-associated bacteria in growing pigs with and without diarrhea differed according to the presence of colonic inflammation
Source: BMC Microbiol. 2023 May 20;23:145. doi: 10.1186/s12866-023-02874-1 (PMC10199627; doi:10.1186/s12866-023-02874-1)
Supplement: Supplementary file 3 — Additional file 3: Table S1. Number of segments and animals in different variables of the dataset for each group. Table S2. Number of samples in each group per analysis. Fig. S1. PCo plot of variance dispersion around the centroids for Bray-Curtis dissimilarity in the whole dataset for digesta vs. mucosal samples (A), in digesta (B), and in mucosal samples (C). P-value below 0.05 indicates lack of homogeneity of variance around centroids. Fig. S2. Differential abundance of phyla (FDR < 0.05) for DiarInfl vs. DiarNoInfl in digesta (A) and in mucus (B). Differentially abundant genera for DiarInfl vs. DiarNoInfl in digesta (C) and in mucus (D). Only genera with FDR ≤ 0.05 and with absolute value of log2FoldChange > 2 are presented. Each genus is colored to its representative phylum and labeled with their correspondent log2FoldChange values. [file 12866_2023_2874_MOESM3_ESM.docx]

**Supplementary materials**

**Tables**

**Table S1:** Number of segments and animals in different variables of the dataset for each group.

| Groups | Segments | Histological samples per segment | Age 8w | Age 11w | Age 12w | SexMale | SexFemale | Pen | Round |
| --- | --- | --- | --- | --- | --- | --- | --- | --- | --- |
| NoDiar | 2 | 5 | 3 | 2 | 0 | 3 | 2 | 4 | 2 |
| DiarNoInfl | 2 | 4 | 6 | 1 | 3 | 2 | 2 | 4 | 2 |
| DiarInfl | 2 | 12 | 2 | 3 | 7 | 8 | 5 | 8 | 2 |

**Table S2:** Number of samples in each group per analysis.

| Analysis | NoDiar | DiarNoInfl | DiarInfl |
| --- | --- | --- | --- |
| Fecal DM | 5 | 4 | 12 |
| Chemical analysis | 10 | 6 | 22 |
| Alpha diversity | 5 | 4 | 12 |
| Beta diversity | 5 | 4 | 12 |
| Differential abundance | 5 | 4 | 12 |

**Figures**

**Fig. S1**


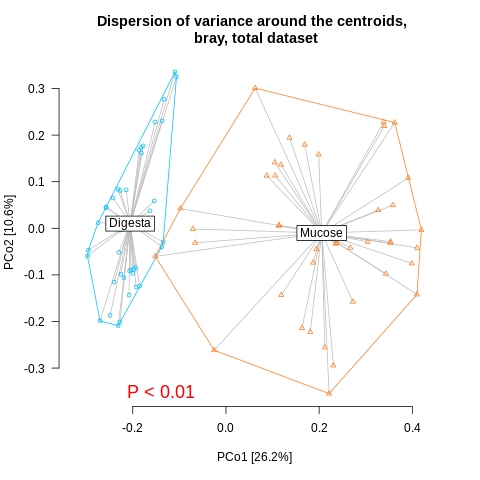


**A**


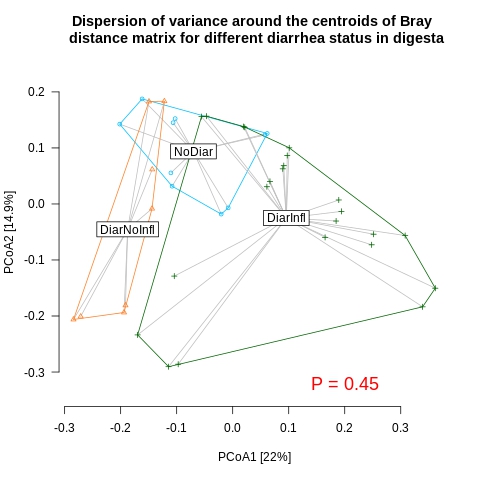


**B**


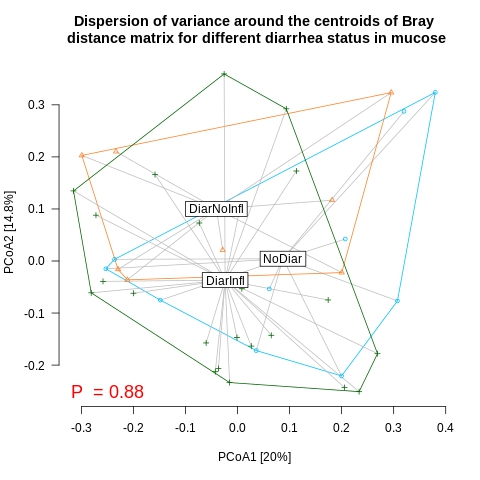


**C**

PCo plot of variance dispersion around the centroids for Bray-Curtis dissimilarity in the whole dataset for digesta vs. mucosal samples (**A**), in digesta (**B**), and in mucosal samples (**C**). P-value below 0.05 indicates lack of homogeneity of variance around centroids.

**Fig. S2**


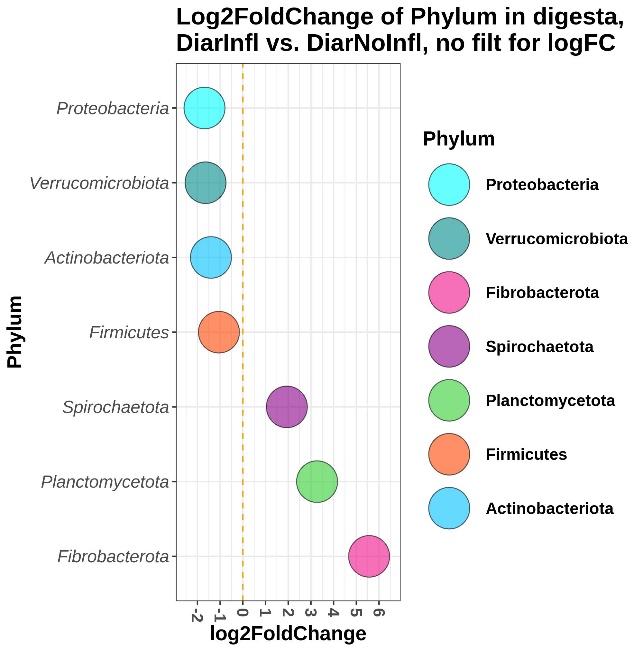


**A**


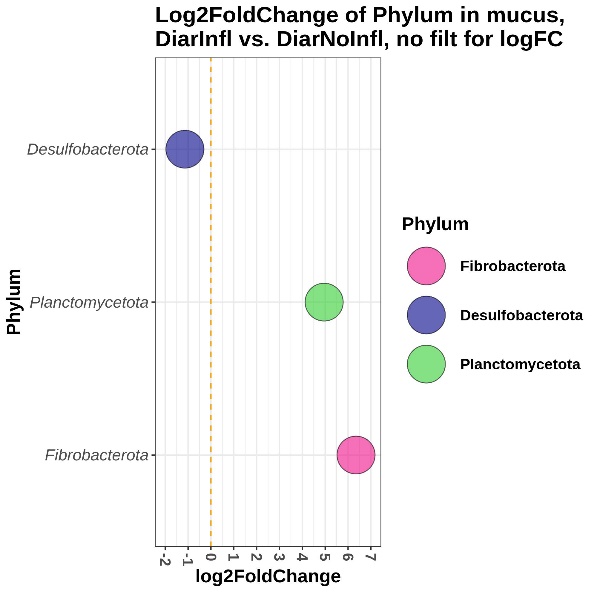


**B**


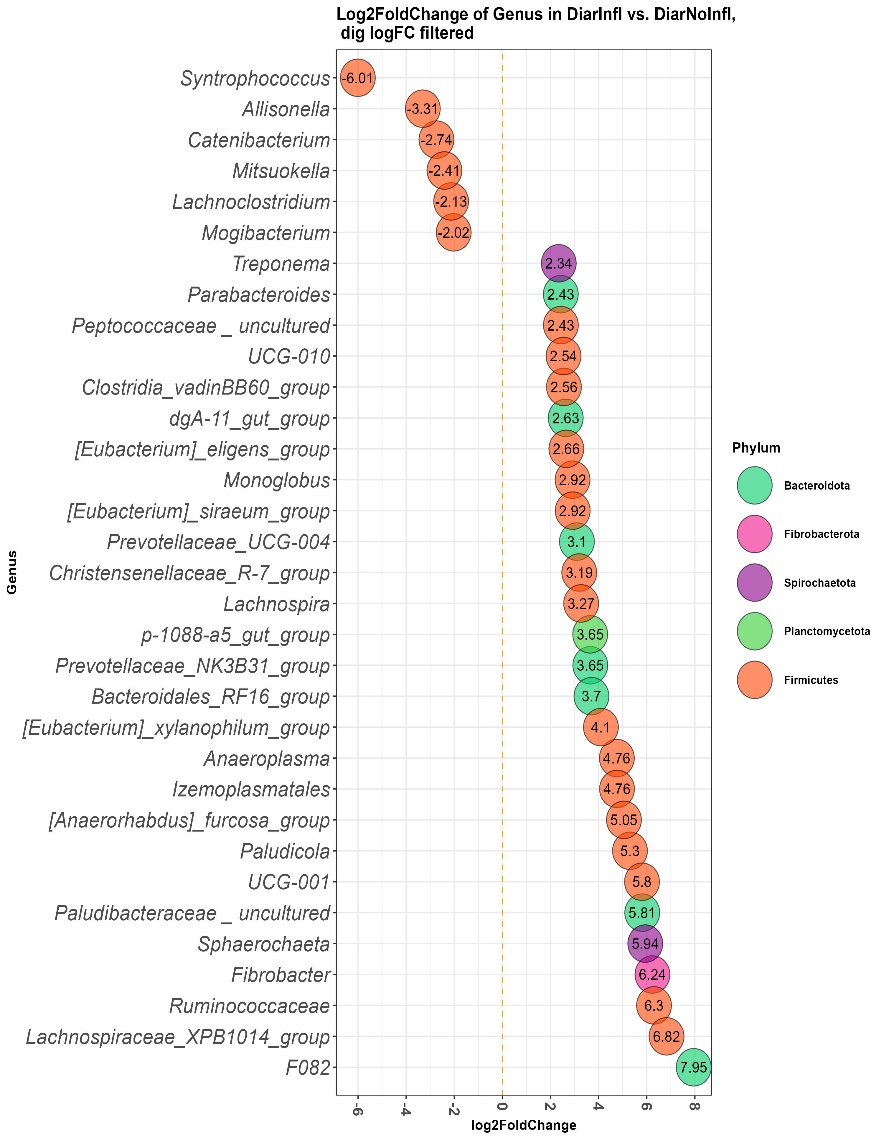

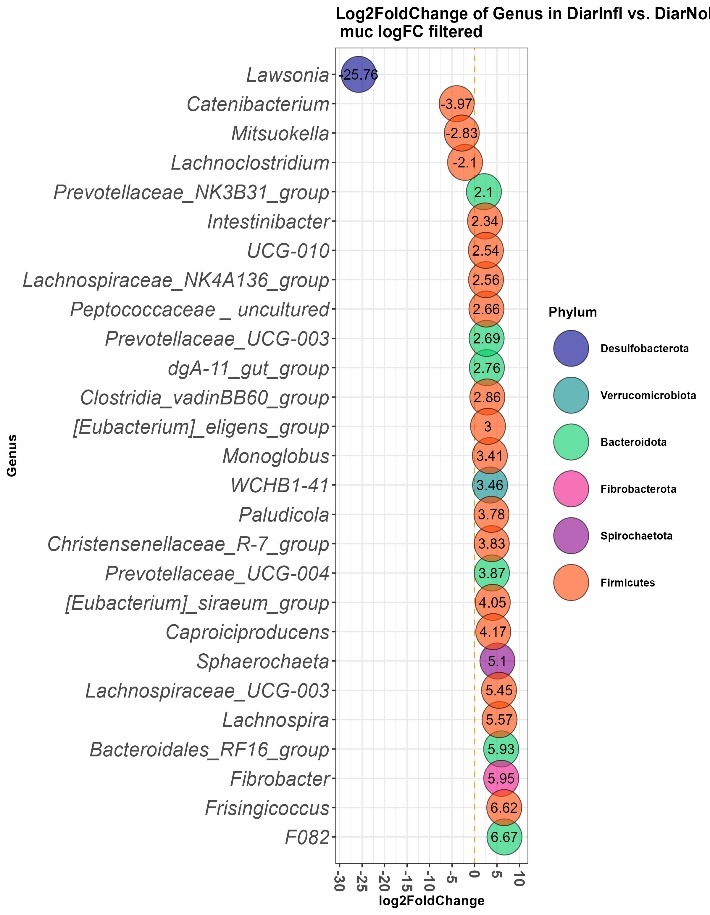


**C**


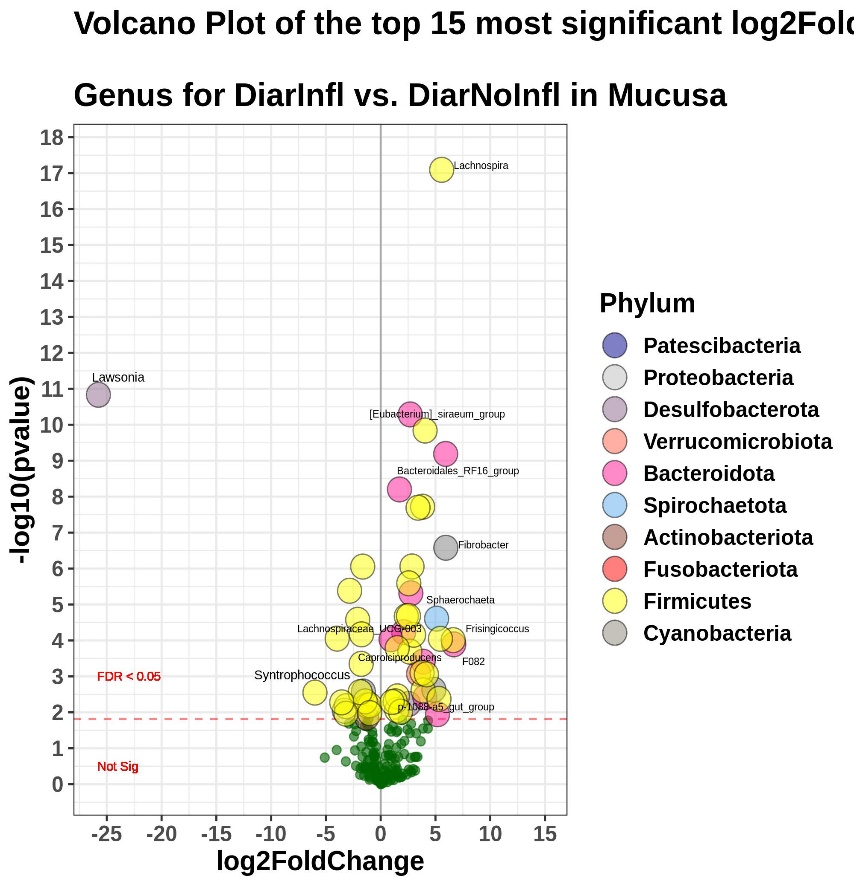


**D**

Differential abundance of phyla (FDR < 0.05) for DiarInfl vs. DiarNoInfl in digesta (**A**) and in mucus (**B**). Differentially abundant genera for DiarInfl vs. DiarNoInfl in digesta (**C**) and in mucus (**D**). Only genera with FDR ≤ 0.05 and with absolute value of log2FoldChange > 2 are presented. Each genus is colored to its representative phylum and labeled with their correspondent log2FoldChange values.
